# The impact of acting training on emotion recognition and expression: a systematic review

**DOI:** 10.3389/fpsyg.2026.1749252

**Published:** 2026-02-16

**Authors:** Vivian Ching-Mei Chu, Ya-Wen Chin, Margot Meng-Cheng Chou, Iris Lavi, Maisie Seaton, Eya Labidi, Jing Yi Ong, Isabelle Feaver, Alison Fang-Wei Wu

**Affiliations:** 1 Department of Drama and Theatre, National Taiwan University, Taipei, Taiwan; 2 Department of Psychology, University of Bath, Bath, United Kingdom; 3 Centre for Longitudinal Studies, Social Research Institute, Institute of Education, University College London, London, United Kingdom

**Keywords:** acting, drama, emotion expression, emotion recognition, intervention, systematic review

## Abstract

**Introduction:**

Acting training focuses on improving actors’ emotion recognition and expression, and it has been used to strengthen individuals’ emotional skills in both artistic and non-artistic contexts. However, when examining the impact of this training on emotion recognition and expression in studies outside the arts, diverse research designs resulted in inconsistent findings or limitations for comparison across studies.

**Method:**

We reviewed and evaluated the research design and evidence.

**Results:**

The 32 included articles show that acting-based interventions have been employed across diverse areas, including clinical, occupational and educational contexts, and these interventions could improve emotion recognition and expression. We examine the possible reasons for the inconsistency found in the literature, evaluating assessment tools and training content.

**Discussion:**

We suggest addressing the underlying mechanism of the acting-based intervention’s impact on emotion recognition and expression skills and subsequently propose future research directions.

**Systematic review Registration:**

Doi: https://doi.org/10.17605/OSF.IO/R2KTB.

## 1 Introduction

Emotion recognition and expression are critical to maintaining individuals’ well-being and social connections (Turner and Turner, 2010; Quaglia et al., 2015; Cordero et al., 2020). Acting is a performance art that requires coordinated use of physical, facial and vocal skills to convey characters’ emotions and narratives, which has been proposed to enhance emotion recognition and expressive abilities [e.g., (Goldstein, 2011; Benitez-Lopez and Ramos-Loyo, 2022)]. However, empirical evidence remains inconsistent, as studies vary widely in research designs, training protocols, and assessment tools, making it difficult to compare results and draw consistent conclusions. To address this gap, the present systematic review examines how acting-based interventions have been implemented across various domains, focusing on research designs, training methods, assessment tools, and, most importantly, whether and how acting-based intervention improves emotional recognition and expression.

Recognising emotions, referring to identifying one’s emotional states by observing visual and auditory nonverbal information (Bänziger, 2014), and expressing emotions, defined as revealing one’s internal emotional processes (Buck, 1985), are essential to human communication and social interaction (Van Kleef and Côté, 2022). Both skills are central components of emotional intelligence (Elfenbein et al., 2010), which is the capability to understand, manage, and express emotions effectively (Salovey and Mayer, 1990; Salovey and Sluyter, 1997). Studies found that individual differences in emotion recognition and expression could result from both the variation of individuals’ previous emotional experiences (Calvo et al., 2014; Israelashvili and Fischer, 2022) and training [e.g., (Benitez-Lopez and Ramos-Loyo, 2022)].

Acting precisely offers individuals a rich repertoire of emotional experiences, along with comprehensive skill development. Acting training is a systematic process of cultivating physical, vocal, imaginative, and psychological abilities that enable performers to portray authentic or dramatic human behaviour and emotion in professional contexts (Hodge, 2010; Evans, 2015). Unlike singing, which focuses on vocal technique and musical interpretation, or dancing, which centres on rhythm and physical precision, acting training integrates voice, body, and mind to convey and regulate emotion within social interaction. It engages both expressive and perceptual components of emotion: actors must not only externalise feelings through movement, tone, and expression but also infer, simulate, and respond to others’ emotional and mental states. This integration of multiple modalities (facial, vocal, and bodily) within coherent, goal-directed exchanges (Berry et al., 2022) closely mirrors real-life interpersonal dynamics, suggesting that its benefits may extend to social and occupational domains (Lewandowska and Węziak-Białowolska, 2023). Moreover, acting can be delivered in diverse forms, from scripted performance to non-verbal improvisation, making it adaptable for people with different abilities or conditions (Cook, 2020). Given its multimodal and flexible nature, acting holds considerable potential beyond the arts, leading Levy (1997, p.72) to describe it as “the best way to educate the emotions”.

Indeed, many acting methodologies worldwide, such as Greek tragedy, Mime, and Stanislavski’s method of acting, focus directly on cultivating emotional skills, including expression, understanding and awareness, in different ways. For example, Greek tragedy, preforming with masks, focuses on the emotional rendering and penetrating power of the actor’s voice, as well as the emotional symbolism of their bodily movements (Dunbar and Harrop, 2018), while Mime, with no vocal expression, places greater emphasis on physical movements and facial expression for portraying different emotions and stories (Evans and Murray, 2023). Moreover, Stanislavski’s training method targets, by drawing on personal memories, to generate authentic emotions that mirror those of the characters, an approach that fosters empathy, accurate expression and emotional awareness (Stanislavski, 1989, 2013, 2016). Despite the different training methods, improving the capabilities of emotion recognition and expression becomes critical in the actors’ training process, regardless of the performance method. As Emunah (2019) suggested, professional actors should be good at understanding and recognising people’s emotions due to their extensive training. In addition, Cameron (1999) found that acting training not only helps actors to accurately capture the characters’ emotions but also improves their emotional awareness and social communication skills, preparing them for day-to-day tasks. The evidence suggests that acting training could help individual enhance their emotional experience.

Accordingly, acting-based intervention has shown positive outcomes (e.g., communication skills, leadership and other socioemotional behaviours) in diverse areas (Milroy, 1982), such as education (e.g., (Hart et al., 2016)), business and management (e.g., (Augustine and Adelman, 1999; Corrigan, 2000)), and clinical settings [e.g., (Moreno, 1934; Emunah, 2019)]. For example, role-playing has been used to simulate workplace conflicts. Through these exercises, participants improve empathy via perspective-taking and develop practical conflict resolution skills applicable to real-world settings (Augustine and Adelman, 1999; Loui, 2009). In addition, studies have shown that acting-based interventions help medical students more accurately identify patients’ emotions and respond with greater sensitivity, empathy, and communication skills in patient-centred scenarios (Kelm et al., 2014; Walsh and Murphy, 2017). Similarly, several studies have linked acting improvisation training to various psychological benefits. These include reduced anxiety and depression in adult psychiatric patients (Krueger et al., 2019), decreased social anxiety in adolescents (Felsman et al., 2019), increased positive affect in social settings, and improved tolerance for uncertainty (Felsman et al., 2019).

Despite these promising applications of acting-based intervention, many uncertainties and gaps remain in its practical implementation and effectiveness. Some studies found that acting-based intervention did not improve socio-emotional skills (Lillard et al., 2013; Lee et al., 2015). For example, Lee et al. (2015) conducted a meta-analysis of 47 quasi-experimental drama-based pedagogy (DBP) intervention studies and found that the results for social outcomes and prosocial behaviours were mixed, with some minor or non-significant effects. The authors attributed this inconsistency largely to methodological heterogeneity across studies, including variation in training designs (e.g., ranging from single sessions to semester-long programmes) and assessment tools (e.g., teacher-rated scales versus standardised questionnaires). Furthermore, McDonald et al. (2020) suggested that limited studies explored how acting interventions may influence emotion skills, especially emotion recognition and expression.

The present review aims to address these gaps by systematically exploring the relationship between acting training and improvements in emotion recognition and expression. It focuses specifically on acting, which uniquely combines multiple modalities of emotional communication within a goal-directed social context, distinguishing it from other performing arts such as dance or singing that emphasise movement or sound alone. To the best of our knowledge, studies have yet to systematically assess the published papers in this area to explore inconsistencies in results and designs across studies. By synthesising evidence across existing studies and comparing methodological characteristics such as publication context, participant features, study design, assessment tools, and training paradigms, this review seeks to clarify whether and how acting training effectively enhances emotional recognition and expression skills, while informing the design of more rigorous and consistent future research.

## 2 Methods

This systematic review followed the Preferred Reporting Items for Systematic Reviews and Meta-Analyses (PRISMA) guidelines (Page et al., 2021). A systematic search was conducted in June 2025 across three major databases: *Scopus*, *Web of Science*, and *PsycINFO*. The search used the following keyword combination:

(*actor* OR ((*acting* OR *actor* OR *theater* OR *theatre* OR *drama* OR “role play*”) AND (*training* OR class*))) AND (“emotion* *recogni*” OR “emotion* *perception*” OR “emotion* identif*” OR “emotion* *understanding*” OR “emotion* process*” OR “affect* recogni*” OR “affect* *perception*” OR “affect* *identif*” OR “affect* *understanding*” OR “affect* process*” OR “mood recogni*” OR “*mood perception*” OR “mood identif*” OR “*mood understanding*” OR “mood process*” OR “feel* recogni*” OR “feel* *perception*” OR “feel* identif*” OR “feel* *understanding*” OR “feel* process*” OR “emotion* generat*” OR “emotion* express*” OR “emotion* produc*” OR “affect* generat*” OR “affect* express*” OR “affect* produc*” OR “mood generat*” OR “mood express*” OR “mood produc*” OR “feel* generat*” OR “feel* express*” OR “feel* produc*”).

**Figure 1:**
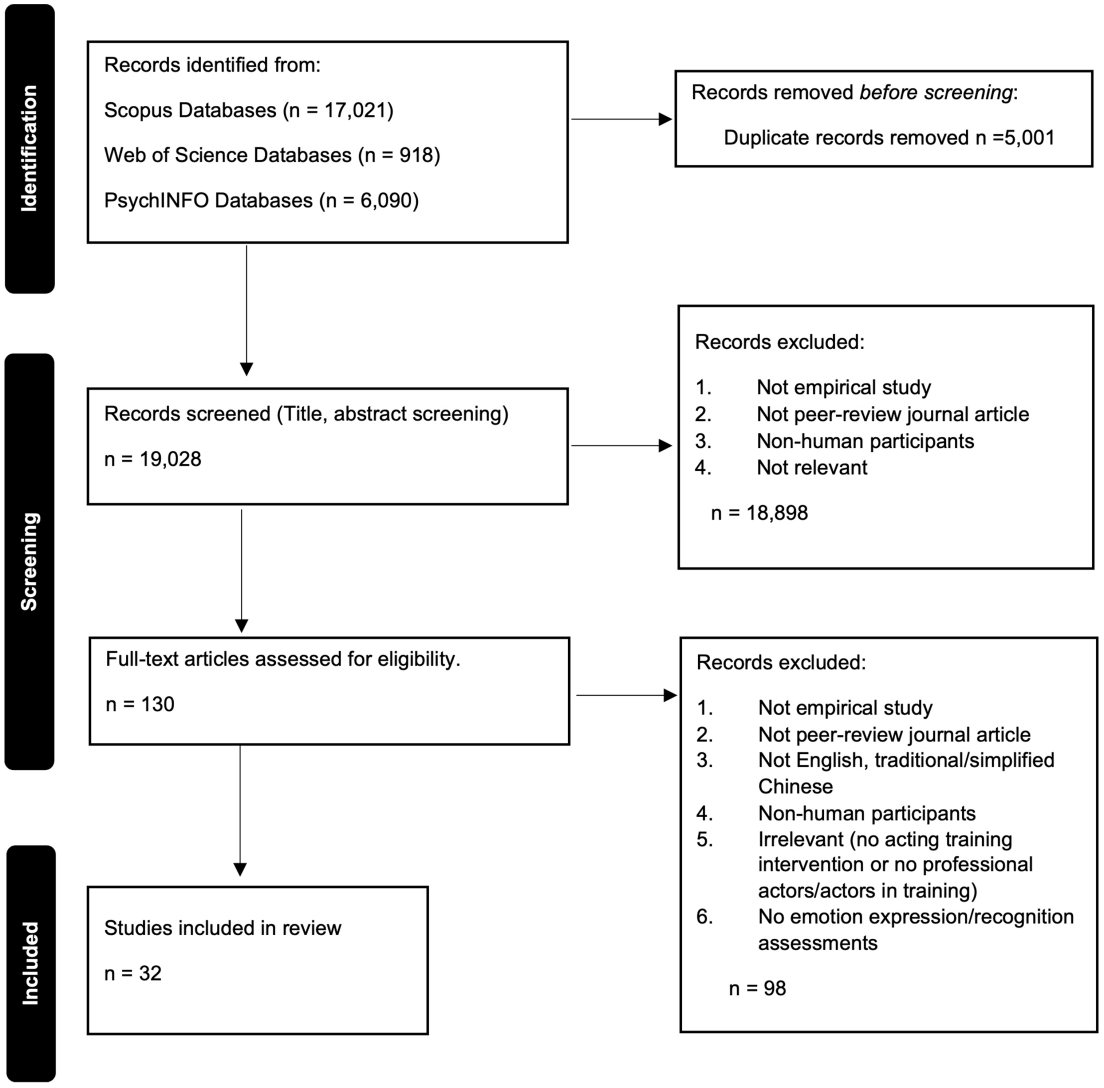
PRISMA flow diagram.

The full search strings were consistently applied across all databases. The number of studies identified from each source is detailed in the PRISMA flow diagram (see Figure 1).

After removing duplicates, YWC, MCC, AFWWU and four volunteered research assistants screened the titles and abstracts using seven inclusion and exclusion criteria: (1) published before 25th June 2025; (2) empirical research; (3) published in a peer-reviewed journal; (4) written in English or Chinese scripts; (5) human participants; (6) having a professional acting expert in acting training interventions or the participants had professional acting training experience; and (7) having emotion recognition or expression skills assessments. To ensure reliability, each article was screened by at least two reviewers. In cases of disagreement, VCMC, YWC, MCC, AFWWU joined the discussion, and a consensus was reached collectively. The same process was applied for full-text screening, which was independently conducted by YWC, MCC, AFWWU and four research assistants. Any discrepancies were again resolved through discussions. The complete screening and selection procedure is illustrated in Figure 1.

### 2.1 Methodology quality assessment—mixed methods appraisal tool

To systematically assess the methodological quality of the included studies, we applied the Mixed Methods Appraisal Tool (MMAT) (Hong et al., 2018). The MMAT is specifically designed to evaluate empirical research across five methodological categories: qualitative, quantitative randomised controlled trials, quantitative nonrandomised, quantitative descriptive, and mixed methods. MMAT evaluates key aspects of study quality, including the clarity of the research question, the appropriateness of the methodological approach, and design-specific quality criteria. This tool is especially suitable for reviews incorporating diverse study designs and has been widely used in recent systematic reviews [e.g., (Willis, 2019; Aithal et al., 2021; Guan and Lo, 2021)].

In addition to the MMAT criteria, research question (RQ) clarity was operationalised as the presence of an explicitly stated research aim or question that could be directly addressed by the reported study design and data. An RQ was considered clear if it specified the phenomenon or outcome of interest and how this would be examined empirically (e.g., through defined outcome measures, pre–post comparisons, or systematic qualitative procedures). Studies were coded as having unclear RQs when aims were described only in broad or exploratory terms without specifying evaluable outcomes or analytic procedures linking the intervention to observed change. This criterion was applied independently of disciplinary orientation and did not penalise methodological complexity or interdisciplinarity.

Each selected article was first classified into one of the five MMAT categories based on its study design. Three reviewers, YWC, MCC and AFWWU, independently assessed each article using two initial screening questions (evaluating the clarity of the research questions and the adequacy of the collected data), followed by five method-specific criteria as specified in the MMAT user guide. Each criterion was rated as “Yes,” “No,” or “Cannot tell,” based strictly on the information reported in the articles and following the MMAT user guide. Any discrepancies in ratings were resolved through discussion among the VCMC, YWC, MCC and AFWWU until consensus was achieved.

## 3 Results

The searches resulted in 24,029 studies with titles and abstracts. 5,001 duplicates were identified and removed. The titles and abstracts of 19,028 studies were screened, and 18,898 were excluded based on the inclusion and exclusion criteria (i.e., not an empirical study, not a peer-reviewed journal article, and/or non-human participants). Subsequently, we retrieved the full texts of 130 remaining studies, and 32 papers were retained for this review after eligibility was assessed, see Table 1.

**Table 1:** Summary of the included papers by groups.

Reference	Location	Age	Gender ratio	N	Participants condition	Design	Training/treatment/intervention	Emotion expression/recognition measures/tasks	Emotion recognition and expression results
Actor/actress versus non-actor/actress									
Gentzler et al. (2020)	USA*	M = 19.86	70% F	284	Theatre major versus some-acting versus non-acting experience group	Group comparison	The study recruited the college students who were theatre majors, but did no mentioned specific training duration	Amsterdam Dynamic Facial Expression Set (van der Schalk et al., 2011); Cognitive reappraisal subscale of the emotion regulation questionnaire (ERQ) (Gross and John, 2003)	Among the three groups, the theatre majors the most accurately identified pride expressions, no-acting group the most accurately identified anger expressions, and no significant differences in the recognition of other emotions (sadness, contempt, fear, disgust, embarrassment, joy, and surprise).
Goldstein (2011)	USA*	13–16 (M = 14)	67.92% F	53	Acting class versus visual arts and music class	Group comparison	One-year acting or arts classes	Cognitive reappraisal subscale of the ERQ, (Gross and John, 2003); Empathic Accuracy Paradigm (Ickes, 2001); Basic empathy scale for adolescents (Jolliffe and Farrington, 2006); Reading the Mind in the Eyes test (RMET) (Baron-Cohen et al., 2001)	No significant differences between the acting group and the other arts groups in their ability in the score of Fiction Emotion Matching, meaning no differences in recognition of sadness and scary.
Höfling et al. (2022)	Germany	undergraduate	100% F	70	Trained actress versus untrained	Group comparison	70 female actors selected from three well-known picture inventories: The Karolinska Directed Emotional Faces, the Warsaw Set of Emotional Facial Expression pictures and the Radboud Faces Database	FaceReader software and aggregated with Observer XT offline (FR; Version 7.1, Noldus Information Technology; Version 12.5, Noldus Information Technology)	The trained actors produced more intense facial expressions compared to the untrained participants.
Jurgens et al. (2015)	Germany	18–35	Not mentioned	288	Actor versus non-actors	Group comparison	The study recruited professional actors through theatre agencies and drama schools, ensuring that they had received formal training and education.	NBS Presentation (Neurobehavioral Systems) was used to present the speech stimuli to the participants and collect their ratings of the specific vocal expression of emotion and authenticity.	Actors’ expressions were recognised more accurately than the non-actors’ expressions, but only for anger, fear, and joy stimuli, not for sadness, play-acted and authentic.
Klinge et al. (2012)	Germany	M = 35	50% F	10 (actors) + previous data: 10 blind, 10 sighted	Professional actors (trained ≥ 3 years, ~4.75 h/week auditory/emotional speech training) versus blind versus sighted controls	Group comparison (fMRI experiment)	Extensive acting training focused on auditory emotional expression (intonation, timbre, vowel quality, intensity) with feedback	Emotion discrimination task (angry, happy, fearful, neutral prosody) (Klinge et al., 2010); Vowel discrimination task (control) (Klinge et al., 2010); fMRI measuring amygdala activation (LeDoux, 2007)	The actors were significantly faster and more accurate than sighted controls, comparable to blind participants in emotion discrimination. However, the amygdala activation did not differ between actors and sighted and was significantly higher in blind participants.
Krahmer and Swerts (2008)	The Netherlands	M = 27 / M = 36.2	59% F / 50% F	70/40	Experienced actors versus inexperienced actors versus non-actors	Group comparison	Actors were either from several theatre companies in Tilburg or were in their final year at Tilburg drama academy. All has between 3 and 25 years of professional experience (M = 11.2 years, SD = 6.5 years).	Participants had to rate the perceived emotional state from the actors’/non-actors’ stimuli on a 7-point valence scale in study 2.	Overall, all stimuli from participants in the data collection experiment (inexperienced actors, experienced actors and non-actors) accurately expressed a positive, negative or neutral emotion. However, surprisingly, expressions from professional actors were found to be more extreme and less realistic than non-professional actors.
Orzechowicz (2008)	USA*	–	Not mentioned	–	No comparison	Only actors	Actors were in acting classes and a theatre.	Over 1000 h of fieldwork participant observation and seven semi-structured interviews were used with novice and semi-professional stage actors to gain insight into their experiences and strategies for managing emotions in the context of their work.	The study emphasised the structured nature of emotion management in the theatre and the resources that enable actors to effectively evoke specific emotions during their performances.
Schmidt et al. (2021)	UK and abroad	UG**	78.2% F	176	Acting versus dancing versus psychology students	Group comparison	The study focused on first-year graduate courses in acting, dance, or psychology.	RMET, (Baron-Cohen et al., 2001); Empathy quotient (EQ) (Baron-Cohen and Wheelwright, 2004); Interpersonal Reactivity Index (IRI) (Davis, 1980)	Acting students showed the highest levels of emotion recognition among the three groups in, based on the results of RMET.
Sun and Okada (2021)	Japan	–	Not mentioned	26	–	Pre and post comparison	Activity training, Meisner technique, is a critical pair work based on Repetition to help participants learn how to interact with their partner in an imaginary situation. Activity training sessions, with a total of 82 sessions, each lasting about 10 min.	The study used a qualitative analysis of the utterances made by actors during the training sessions to identify changes in their attentional focus and emotional expression over time. They also conducted interviews with two actors to gain insight into their subjective experiences of the training.	By concentrating on their partners rather than themselves, actors were able to perform actions and responses in real time in specific situations, which led to real emotional involvement, rather than expressions.
Clinical context									
Abdulhaq et al. (2025)	Jordan	9–10	25% F	12	Behavioural and/or emotional difficulties (e.g., hyperactivity, anxiety, withdrawal, or peer issues).	Pre and post comparison	Ten weekly integrative visual arts therapy (drawing, painting and sculpting) and dramatherapy techniques (role-play, storytelling and movement-based games). The sessions were delivered by certified art therapists with additional training in drama-based therapeutic method with two trained observers to record behaviours The sessions were structured with an opening (breathing exercise), main activities and closing reflection.	Structured behavioural observations, including quantitative checklists on frequency of participation, instances of emotional expression, and quantitative notes on emotional breakthroughs or resistance.	Noticeable transformation in increased children’s participation, improved emotional articulation (confident use of body or facial expression), and better peer interactions.
Agnihotri et al. (2012)	Canada	16&17	100% F	2	Severe childhood brain disorder	Pre and post comparison	The theatre skills training, including improvisation, vocal exercises, and role-playing, were designed to facilitate social skills and participation for youth living with childhood brain disorder. The training was led by three professional theatre artists and three master’s level occupational therapy students. The intervention lasted for 3 weeks, with daily sessions of 4 h each.	Emotional discrimination task; BarOn Emotional Quotient Inventory (Bar-On and Parker, 2000); Canadian occupational performance measures (Law et al., 1994); Framework Analytic Approach (Ritchie and Spencer, 2002)	The intervention had a significant impact on the participants’ ability to identify emotional expressions, such as angry, sad, and happy, from static images of faces, coupled with decreases in reaction time from pre- to post-intervention assessments and follow-up.
Agnihotri et al. (2014)	Canada	13–16	20% F	5	Acquired brain injury	Pre and post comparison; group comparison	The interventions involved theatre-based activities such as vocal exercises, improvisational acting, and story building, among others. It lasted for 4 weeks, with daily sessions of 4 hours each	Emotion discrimination task	The improvements were observed in accuracy and reaction time in identifying emotional expressions and emotional regulation
Corbett et al. (2011)	USA	6–17 Aut M = 11.30 (SD = 3.98) Typ M = 13.86 (SD = 3.49)	31.25% F	16	Autism Spectrum or pervasive developmental disorder versus typically developing children	Pre and post comparison; Group comparison	Social Emotional NeuroScience Endocrinology (SENSE) theatre program involved participants attending majority of 38 rehearsals and 6 performance dates over 3 months.	NEPSY Affect Recognition (Korkman et al., 2007)	The identification of facial expressions did not reach statistical significance
Eschenauer et al. (2023)	France	10.5	0% F	1	Neurodevelopmental disorder (NDD) characterised by a limitation in cognitive functioning and adaptation capacities.	Pre and post comparison	Performative theatre workshops divided into 8 sessions of 2 h over 4 months. Complimented by classroom language activities twice a week in sessions of 30 min–1 h.	Cognitive-emotional skills (LEAS-C) (Bajgar et al., 2005).	Analysis showed a clear progression in emotional awareness of self and others
Horwitz et al. (2010)	Sweden	M = 53	100% F	7	Fibromyalgia	Pre and post comparison	Theatre-related method (TRM), 3-month and 2-h per week, included training in body and voice expression and acting out a drama onstage with professional actors.	Video interpretation technique: patient interpreted herself as she appeared on the videotape and used a 10-point scale to evaluate the intensity of emotional expression in each film.	Theatre can be a useful tool for fibromyalgia patients to express their emotions and improve their self-rated health.
Keightley et al. (2018)	Canada	9–14	Not mentioned	3	Fetal alcohol spectrum disorder (FASD)-related diagnosis	Pre and post comparison	The arts-based theatre training led by a professional theatre artist and educator. It included voice work, breathing, movement, physical warm-up, character development, three-dimensional awareness, group dynamics, story development, mask work, circus skills training, and clowning. It processed 5 days a week for 4 h over a period of 4 weeks	A self-reported scale of 1 to 10 to be able to communicate their feelings and emotional needs.	The intervention help express their feelings and improved their emotion expression
Mele et al. (2019)	Italy	47–49	28.7% / 31.7% F	42/26	Schizophrenia	Pre and post comparison; group comparison	Theatre activities, each patient was supported by a professional educator and consisted of regular participation in rehearsals for the shows and public performances in regional theatres, as well as in national and international tours	To show participants a series of faces and ask them to identify the emotion being expressed.	The results suggest that drama therapy may be an effective intervention for improving social cognition deficits in individuals with schizophrenia, specifically in the area of facial expression categorization.
Pires et al. (2020)	Portugal	16–18 (M = 16.6; SD = 0.8).	40% F	5	Anxiety problems, specifically translated in interpersonal relationships	Pre and post comparison	Therapeutic Mask intervention tool for psychodrama group which was led by an experienced and trained director. It lasted 8 weekly and 90 min for each.	Helpful Aspects of Therapy (HAT) (Llewelyn et al., 1988) and the Clinical Change Interview (CCI) (Elliott et al., 2001).	The adolescents reported improvements in emotional expression and regulation as a result of the intervention.
Robinson and Kalawski (2022)	UK	16–29	45.5% F	11	Autism spectrum or Asperger Syndrome	Pre and post comparison	The step-out technique in emotion-focused therapy is a tool used in emotion regulation that involves ending each emotional reproduction by at least three slow, regular, and deep, full breathing cycles followed by a total relaxation of the facial muscles and a change in posture.	Client-emotional processing scale for autism spectrum (Robinson and Elliott, 2016)	The intervention helped participants with autistic process shift their attention from an externalised to an internalised process, and to recognise, express, and regulate their internal states.
Tang et al. (2020)	USA	15–25	46% F	36	Clinical risk (CR) for psychosis	Pre and post comparison; group comparison	Theatre Improvisation Training to Promote Social Cognition (TIPS) involves 18 weekly 2-h group sessions led by a theatre director and actor-assistant. Participants engage in collaborative acting and improvisation exercises of varying degrees of complexity, intensity of affect, and interpersonal demand.	Penn computerized neurocognitive battery (CNB) (Moore et al., 2015)	TIPS has no effect on facial emotion processing
van Huisstede et al. (2024)	USA	3–5 (M = 50.71 months; SD = 6.44)	43% F	196	Preschoolers, 10% identified as having special needs or disabilities	Pre and post comparison; group comparison	Drama-based instruction (DBI) as part of the EYEPlay program paired professional teaching artists with preschool teachers. Students were expected to experience at least 18 DBI story times but only participated in at least 15 due to the COVID-19 pandemic. Each session begins with discussing drama word or social phrase and encourages children to embody story characters by considering their thoughts and feelings, pantomiming actions and generating new ideas. Children are also given the opportunity to respond physically to reflection questions.	Story Recall Measure (SRM), including the frequency of target emotion words used. Video and audio recording were coded using Embodied Coding System (ECS) (Bernstein et al., 2024)	Intervention children show increased embodied behaviour, especially gestures than the control group. Younger children in intervention also used more emotion words in prompted recall compared to control group. However, there were no overall group differences in free or prompted recall of character feeling states. Embodied behaviours, including facial expressions and vocal changes were positively associated with emotion word production across groups.
Occupational scenes									
Albal et al. (2021)	Turkey	M = 35.54 (SD = 9.03)	96% F	26	Nurses working at the acute and chronic psychiatric clinics of a psychiatric hospital	Group comparison	The study used psychodrama to help the experimental group gain role flexibility, understand each other’s emotions, recognize their own emotions through catharsis, and develop communication skills. The psychodrama lasted 2 h on each Thursday for 8 weeks. In total, 16 h were led by a certified psychodrama therapist and a co-therapist	Levels of Emotional Awareness Scale (LEAS) (Kuzucu, 2008);	Compared with the control group, the psychodrama helped the experiment group better recognise and express their emotions, understand themselves and others, and connect with their own feelings and thoughts better.
Briones et al. (2022)	Spain	M = 18.82 (SD = 3.07) M = 19.16 (SD = 3.46)	S1:75.7%F S2:77.6%F	S1:280 S2:626	Initial / student teacher	Pre and post comparison	Used Creative Drama (CD) and Forum Theatre (FT) to work on conflicts faced by teachers and to enhance students’ social, emotional, and ethical (SEE) skills, which was a one-semester course (approximately 3 months).	Cognitive and affective empathy (Pérez and Pinto, 2009)	The use of CD and FT can have a positive impact on the empathy for the factor of emotional understanding.
Del Vecchio et al. (2022)	USA	Freshmen and Sophomore	Not mentioned	225	Nursing students	Pre and post comparison	2 workshops (non-verbal & verbal communication) were based on classic acting exercises and designed to develop core communication and interpersonal skills. The workshop varied in length from 30 to 90 min, as part of the nursing courses in which students learn to interview patients.	5-point Likert scale (sensitivity to emotions expressed by others).	Greater than 85% agreed or strongly agreed that the workshop developed their sensitivity to emotions expressed by others
Firing et al. (2022)	Norway	–	Not mentioned	14	Royal Norwegian Air Force Academy students	Pre and post comparison	Theatre-based leadership development programs: (1) Yourself, (2) Relational and social knowledge, and (3) Leadership in the Air Force. It was a nine-week course.	Eight in-depth interviews	The cadets acquired experiences of how emotions can influence followers in relational contexts, and their acting involved emotional awareness and regulation by means of relational processes with their co-actors and the audience.
Sisman and Buzlu (2022)	Turkey	19–21	89.2% F	120	Nursing students	Group comparison	Emotion-Focused Training Program (EFTP): psychodrama techniques was used, such as sociometry and doubling, role-play, and mirroring. The intervention consisted of 10 sessions, each lasting 1.5–2 h, once a week.	The Emotional Expression Scale (Kuzucu, 2011): verbally and nonverbally. The Levels of Emotional Awareness Scale	EFTP improved emotion expression skills. Moreover, improved emotion recognition and expression persisted 6 months post-program during the follow-up assessment. EFTP also improved emotional awareness skills.
Children/school students									
Celume et al. (2020)	France	9–11	Not mentioned	126	–	Pre and post comparison	Drama Pedagogy Training (DPT): children were asked to create and play a scene with their faces and bodies while the other half of the class had to guess what was going on in the scene, giving details and explanations of their guessing. Collective supportive game (CSG, control group’s active intervention): children are divided into two groups, one half has to deliver a message (scarf) and combatants have to avoid the message to be delivered by stealing the message. Total 6 sessions and each lasted 60–70 min.	Reading the Mind in the Eyes Test (RMET-G), the ability to infer mental states from facial expressions (Baron-Cohen et al., 1997)	The intervention can promote children’s socio-emotional competencies, such as theory of mind and collaborative behaviour.
Celume and Zenasni (2022)	France	9–10, 4th grader (M = 9.78)	56% F	9	–	Pre and post comparison	DPT: Each couple of children are partners in a robbery and have to decide if betray his/her partner to save him/herself or not. The objective of the game is to stay in prison the less time that is possible. At the end of each session, a facilitator asked guided questions to help participants thinking. Total 4 sessions and each lasted 60–70 min.	RMET-G, French version (Baron-Cohen et al., 1997)	A positive effect of the drama pedagogy program on the children’s mood but did not show significant improvement in ToM after the training. Also, the observation test showed that the participants spent most of their time discussing socio-emotional themes.
Gürle (2018)	Turkey	10–12 years old	50% F	10	Syrian refugee children	Pre and post comparison	The intervention was a creative arts and drama workshop, made up of 5 2-h sessions over 2 weeks, involving storytelling, image theatre, improvisation, role play, puppetry, miming, masques and character work. It was led by one lead-facilitator and two assistants and aimed to improve identification and expression of emotions and their communication skills.	The study was an exploratory study that used participant observations of what the children drew and responded to certain statements.	The intervention provided evidence for improved ability to empathise with others and enhanced emotional expression through words or drawings.
Kosma et al. (2024)	USA	Undergraduate M = 20.43	85.71% F	7	–	Pre and post comparison	Undergraduate students enrolled in one semester long physical theatre (PT) class. Two 1.5 h sessions a week. Participants were interviewed at the start of the semester and at the end.	Audiotaped, structured in-person interviews by first author. Themes and subthemes were identified in the transcripts.	Participants stated that PT allowed them to unlock their bodies and freely express their emotions. Some participants stated they can express more emotions through movement then they could before PT (not just happy and sad).
Nikiforidou and Stack (2019)	UK	3–4	Not mentioned	33	–	Pre and post comparison	Two conditions: empathic and non-empathic. Each session has three phases: Phase 1 was the read-aloud of the story and phase 2 was the enactment of the story through the perspective of one character per session; namely, session 1: little red riding hood, session 2: grandma, session 3: woodcutter and session 4: wolf. During phase 2, the facilitator would encourage the narration and acting out of the story by addressing for each character four critical moments/dilemmas (Table 1). In phase 3, the children and the facilitator would discuss and reconsider aspects of the story through discussion and free play with figurines of the story-characters. Total 4 sessions and each lasted 30 min.	Video recordings: observational instrument of motor skills (OSMOS) (Castañer et al., 2009)	Children who participated in the empathic condition would use emotions more frequently in their narratives compared to children who in the non-empathic condition. Children were able to verbalise or show more emotion expression when they were encouraged by the facilitators.
Rousseau et al. (2012)	Canada	12–18	55% F	55	–	Pre and post comparison; group comparison	Each session includes a warm-up period composed of theatrical exercises and of a language awareness activity which also uses dramatization. In the second half of the sessions, stories contributed by the youth are explored and played out in small groups, using either nonverbal expression or the spoken language of their choice. Total 12 sessions (1 session per week) and each lasted 90 min.	Participant observations and feedback from teachers and students	Facilitated their expression of feeling

Further detailed information is included in Supplementary Table 5. * Did not specify the location. We used the first authors’ location as where the research was conducted. ** Meaning undergraduate students.

In the following sections, four themes are presented: (1) study characteristics, summarising the research designs of selected articles, including countries and years of publication, participant features, experimental design, assessment tools, and intervention content, (2) quality assessment, evaluating the methodological quality of selected articles, (3) areas of application, outlining the domains in which acting training has been used to improve individuals’ skills or abilities, and (4) the effects of acting training, synthesising the evidence on its impact on emotion recognition, expression, and other related skills.

### 3.1 Study characteristics

**Figure 2:**
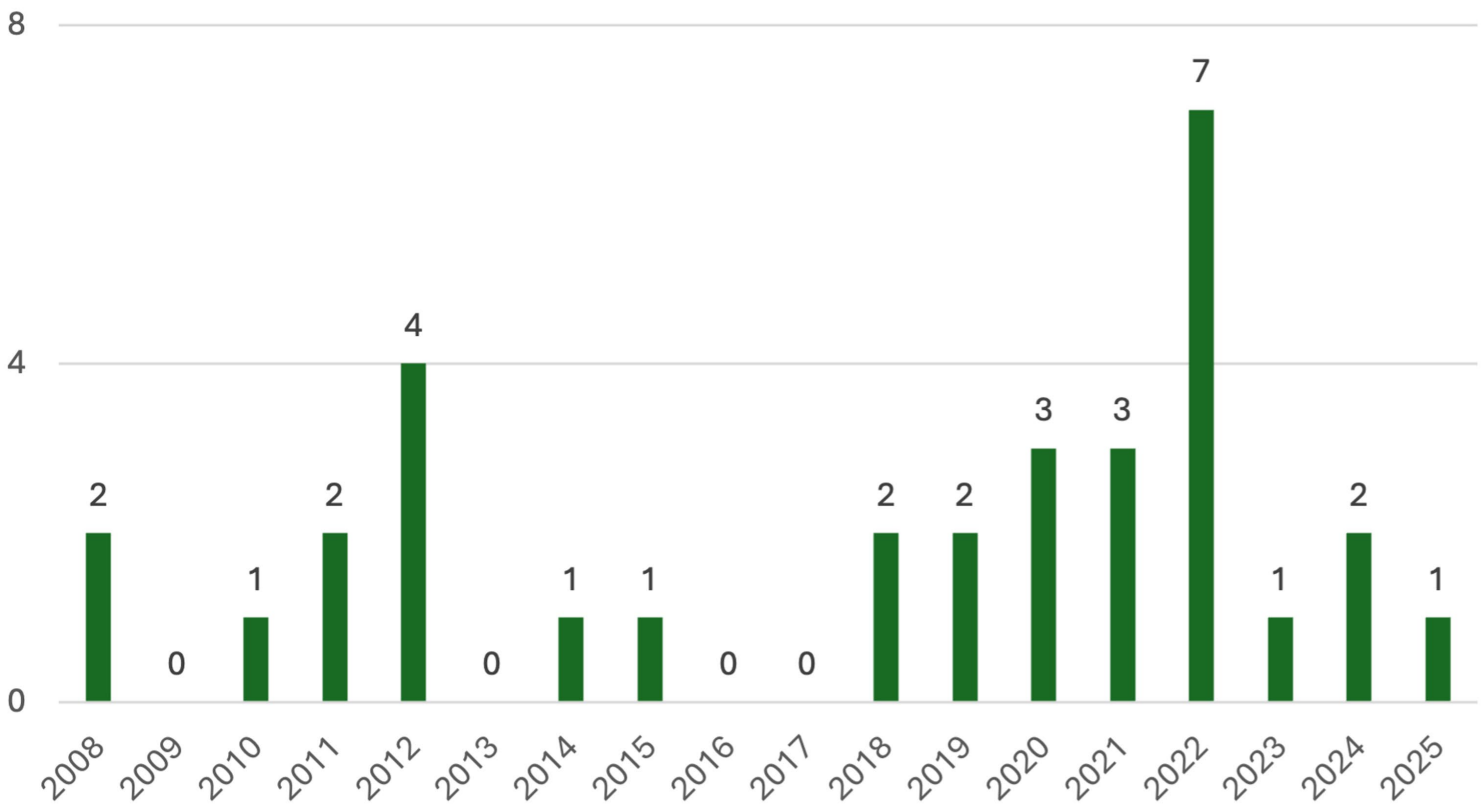
Years of selected articles were published before June 2025.

Most studies were conducted in European or North American countries (i.e., the U. K., France, Sweden, Norway, Italy, Spain, Portugal, Turkey, the U.S.A., and Canada), except one, which was conducted in Japan (Sun and Okada, 2021) and one which was conducted in Jordan (Abdulhaq et al., 2025). This shows that the utilisation of acting-based intervention for emotional improvement is limited in areas outside Europe or North America. The number of publications has increased in recent years, showing an upward trend after 2018 (see Figure 2). However, there was a decline after 2022, which may reflect the impact of the COVID-19 pandemic, as restrictions limited the delivery of in-person interventions.

The selected articles varied in sample size, ranging from one to 626, and included diverse participant characteristics, such as age, gender distribution, and health conditions. The participants’ ages ranged from 3 to 50s in these articles. Females were more likely than males to be involved in this type of research. Specifically, 24 articles provided detailed gender information: 10 articles recruited only females or more females than males, eight reports had approximately equal numbers of males and females (around 40–60%), and six articles recruited more males than females. This showed that men could be underrepresented in the target population.

Table 2 shows the counts of selected articles that measured emotion recognition, expression, or both. Emotion expression was less explored than emotion recognition in the selected articles. Among these 32 papers, eight employed qualitative assessment tools, 17 utilised quantitative measures, and seven incorporated a mixed-method approach. Emotion recognition mainly was measured with quantitative tools (18 of 24), whereas more qualitative (nine) than quantitative (seven) tools were used to assess emotion expression. Some studies adapted research tools through localisation (e.g., translation into the local language or alignment with cultural contexts) and utilised modified versions for their investigations. Lacking localised assessment tools might limit the execution of relevant research outside Europe and North America. The research tools are detailed in Supplementary Table 1.

**Table 2:** Emotional ability training target: number of articles that examine the improvement of emotion recognition, emotion expression, or both.

Emotional ability training target	Counts	Selected articles
Emotion “recognition”	10	Horwitz et al. (2010), Corbett et al. (2011), Goldstein (2011), Klinge et al. (2012), Agnihotri et al. (2014), Celume et al. (2020), Gentzler et al. (2020), Schmidt et al. (2021), Briones et al. (2022), Eschenauer et al. (2023)
Emotion “expression”	8	Krahmer and Swerts (2008), Rousseau et al. (2012), Jurgens et al. (2015), Pires et al. (2020), Höfling et al. (2022), Robinson and Kalawski (2022), Kosma et al. (2024), Abdulhaq et al. (2025)
Emotion “recognition” and “expression”	14	Orzechowicz (2008), Agnihotri et al. (2012), Gürle (2018), Keightley et al. (2018), Mele et al. (2019), Nikiforidou and Stack (2019), Tang et al. (2020), Albal et al. (2021), Sun and Okada (2021), Celume and Zenasni (2022), Del Vecchio et al. (2022), Firing et al. (2022), Sisman and Buzlu (2022), van Huisstede et al. (2024)

In addition to assessing emotion recognition and expression abilities, the selected articles also evaluated the effectiveness of acting training interventions on various capabilities. We categorised these additional abilities into six major fields, including: (1) social skills (e.g., interaction, communication and collaboration.); (2) physical/mental well-being; (3) psychological abilities (e.g., emotion skills beyond recognition and expression, empathy, self-esteem, leadership, creativity); (4) effectiveness of specific topics (e.g., instructional resources, skill application, community activities, and therapy), and (5) others (e.g., acoustic analysis, demographic characteristics, and promigratory experience). Supplementary Table 2 shows detailed content and related research tools in each field. These also reveal that acting training was applied to help improve other skills.

Of the 32 papers, 23 employed acting training in their research, and their interventions varied in terms of timing and activities. Given that the time features, such as duration, frequency, and cumulative total training hours during the training period, are critical to the effectiveness of acting training (Goldstein et al., 2020), we summarised these features of the training in these articles. Our results revealed various time features: 70% of these 23 papers conducted acting training interventions over 8 weeks. Nearly 67% (16 articles) of these 23 papers involved acting training ten times or more, six articles conducted two to nine times, and only one paper conducted a single workshop (Robinson and Kalawski, 2022). As for the total training hours, approximately 60% (14 articles) were conducted for 10–30 h; three involved less than 10 h, while six did not provide a clear indication. The detailed training differences for each categorisation are presented in Supplementary Table 3.

In the 23 papers conducting acting training, we categorised the content of their respective acting training into three main groups: (1) non-verbal/physical training (e.g., developing the performer’s voice and body, including physical warm-up, voice work, breathing, movement, and three-dimensional awareness); (2) text/role/line training (e.g., emphasising the development of performer’s cognitive abilities, such as group dynamics, story development, character development, script analysis, and writing skills), and (3) specific acting training method (e.g., established performance methods or authoritative books on complete training systems, including clowning, mask work, psychodrama, drama pedagogy training, and Spanish program “programa juego.”). Of these 23 papers, two listed only the activities they applied, six named all the processes and provided examples with details, and 15 explained the details of the training process or provided reference sources. Detailed information on the acting training for each group can be found in Supplementary Table 4.

### 3.2 Quality evaluation results (MMAT)

All 32 included studies passed the two initial screening questions and were categorised into five research design types according to the MMAT criteria: eight qualitative studies, four randomised controlled trials, 12 quantitative non-randomised studies, one quantitative descriptive study, and seven as mixed methods studies, see Table 3.

**Table 3:** MMAT.

1. Qualitative studies	S1. Are there clear research questions?	S2. Do the collected data allow to address the research questions?	1.1 Is the qualitative approach appropriate to answer the research question?	1.2 Are the qualitative data collection methods adequate to address the research question?	1.3 Are the findings adequately derived from the data?	1.4 Is the interpretation of results sufficiently substantiated by data?	1.5 Is there coherence between qualitative data sources, collection, analysis and interpretation?
Firing et al. (2022)	Yes	Yes	Yes	Yes	Yes	Yes	Yes
Gürle (2018)	No	No	/	/	/	/	/
Keightley et al. (2018)	Yes	Yes	Yes	Yes	Yes	Yes	Yes
Kosma et al. (2024)	Yes	Yes	Yes	Yes	Yes	Yes	Yes
Orzechowicz (2008)	Yes	Yes	Yes	Yes	Yes	Yes	Yes
Pires et al. (2020)	Yes	Yes	Yes	Yes	Yes	Yes	Yes
Robinson and Kalawski (2022)	Yes	Yes	Yes	Yes	Yes	Yes	Yes
Sun and Okada (2021)	Yes	Yes	Yes	Yes	Yes	Yes	Yes
2. Quantitative randomized controlled trials	S1. Are there clear research questions?	S2. Do the collected data allow to address the research questions?	2.1 Was the randomization appropriately performed?	2.2 Were the groups comparable at baseline?	2.3 Were there complete outcome data?	2.4 Were outcome assessors blinded to the intervention provided?	2.5 Did the participants adhere to the assigned intervention?
Albal et al. (2021)	Yes	Yes	Cannot tell	Yes	Yes	Yes	Yes
Celume et al. (2020)	Yes	Yes	Yes	No	Yes	Cannot tell	Yes
Sisman and Buzlu (2022)	Yes	Yes	Yes	Yes	Yes	Cannot tell	Yes
van Huisstede et al. (2024)	Yes	Yes	Yes	Yes	Yes	Cannot tell	Yes
3. Quantitative non-randomized studies	S1. Are there clear research questions?	S2. Do the collected data allow to address the research questions?	3.1 Are participants representative of the target population?	3.2 Are measurements appropriate regarding both the outcome and intervention (or exposure)?	3.3 Are there complete outcome data?	3.4 Are the confounders accounted for in the design and analysis?	3.5 During the study period, is the intervention administered (or exposure occurred) as intended?
Corbett et al. (2011)	Yes	Yes	No	Yes	Yes	No	Yes
Gentzler et al. (2020)	Yes	Yes	Cannot tell	Yes	Yes	No	Yes
Goldstein (2011)	Yes	Yes	Cannot tell	Yes	Yes	No	Yes
Höfling et al. (2022)	Yes	Yes	Cannot tell	Yes	Yes	No	Yes
Horwitz et al. (2010)	Yes	Yes	No	Yes	Yes	No	Yes
Jurgens et al. (2015)	Yes	Yes	No	Yes	Yes	No	Yes
Klinge et al. (2012)	Yes	Yes	No	Yes	Yes	Yes	Yes
Krahmer and Swerts (2008)	Yes	Yes	No	Yes	Yes	Yes	Yes
Mele et al. (2019)	Yes	Yes	Cannot tell	Yes	Yes	No	Yes
Rousseau et al. (2012)	Yes	Yes	Cannot tell	Yes	Yes	No	Yes
Schmidt et al. (2021)	Yes	Yes	Cannot tell	Yes	Yes	No	Yes
Tang et al. (2020)	Yes	Yes	Yes	Yes	Yes	Yes	Yes
4. Quantitative descriptive studies	S1. Are there clear research questions?	S2. Do the collected data allow to address the research questions?	4.1 Is the sampling strategy relevant to address the research question?	4.2 Is the sample representative of the target population?	4.3 Are the measurements appropriate?	4.4 Is the risk of nonresponse bias low?	4.5 Is the statistical analysis appropriate to answer the research question?
Del Vecchio et al. (2022)	Yes	Yes	Yes	Cannot tell	Yes	Yes	Yes
5. Mixed methods studies	S1. Are there clear research questions?	S2. Do the collected data allow to address the research questions?	5.1 Is there an adequate rationale for using a mixed methods design to address the research question?	5.2 Are the different components of the study effectively integrated to answer the research question?	5.3 Are the outputs of the integration of qualitative and quantitative components adequately interpreted?	5.4 Are divergences and inconsistencies between quantitative and qualitative results adequately addressed?	5.5 Do the different components of the study adhere to the quality criteria of each tradition of the methods involved?
Abdulhaq et al. (2025)	No	No	/	/	/	/	/
Agnihotri et al. (2012)	Yes	Yes	Yes	Yes	Yes	Yes	Yes
Agnihotri et al. (2014)	Yes	Yes	Yes	Yes	Yes	Yes	Yes
Briones et al. (2022)	Yes	Yes	Yes	Yes	Yes	Cannot tell	Yes
Celume and Zenasni (2022)	Yes	Yes	Yes	Yes	Yes	Yes	Yes
Eschenauer et al. (2023)	No	No	/	/	/	/	/
Nikiforidou and Stack (2019)	Yes	Yes	No	Yes	Yes	Yes	Yes

Among the eight qualitative studies, all met the five MMAT criteria with a “Yes” rating, except Gürle (2018), which had an unclear research question, making it difficult to determine how the collected data addressed the study objectives. For the 12 quantitative non-randomised studies, one received a “Yes” rating across all MMAT criteria. The remaining studies were mostly rated as “No” or “Cannot tell,” particularly regarding whether participants were representative of the target population and whether potential confounders were accounted for in the study design or analysis. Specifically, in terms of representativeness, many studies did not specify the intended target population or describe its characteristics, making it difficult to determine how well the study samples reflected that population. The single quantitative descriptive study was rated “Cannot tell” on the sample’s representativeness, showing similar issues to the quantitative non-randomised studies. Among the seven mixed methods studies, one received a “No” on one criterion, another a “Cannot tell,” two lacked clear research questions, while the remaining three met all five criteria with “Yes” ratings. Overall, more than half of the selected studies (18) received either all “Yes” ratings or only one “No” or “Cannot tell,” suggesting that, despite some methodological limitations, the overall quality of evidence was generally acceptable and provides a reasonable basis for interpretation. Notably, the three studies rated as having lower research question clarity and methodological appropriateness were not excluded from the review and were retained because they contributed relevant findings. Their lower ratings reflect the fact that aims were framed in broad, exploratory terms without clearly specified evaluable outcomes or analytic procedures linking the intervention to observed change. The research quality evaluation is reported to ensure transparency for readers, with lower ratings indicating limitations in evaluability rather than limited relevance or practical value.

### 3.3 Areas of application

After the selection process, as expected, acting training has been applied in diverse areas beyond the art field. For the purpose of synthesis and comparison, we identified four areas of applications based on the primary research context, that is, the main setting or population to which each study was principally oriented: (1) nine professional training of actors, (2) 12 clinical applications, (3) five occupational training, and (4) six education and development. Notably, some studies exhibited characteristics spanning multiple domains (i.e., school-based interventions targeting clinical populations). Where this occurred, studies were grouped according to their primary research orientation, informed by stated aims, target population, and the focus of reported outcomes, with categories serving as analytical anchors rather than mutually exclusive labels. The following sections present detailed information, including acting experiences, the participants’ features, such as health conditions or occupations, and interventions for each group.

#### 3.3.1 Professional training of actors

Nine articles conducted comparative analyses of professional versus non-professional actors (see Table 1). Professionals from schools (eight articles), acting agencies [one article, (Jurgens et al., 2015)], and theatres [two articles, (Krahmer and Swerts, 2008; Orzechowicz, 2008)]; were recruited. This showed a potential preference for school participants, possibly due to the convenience of finding acting-major students for the studies. The actors’ acting training experience varied, with one study indicating an average of approximately 14 h, two reporting 1 year, one reporting over 3 years, and five studies not providing the relevant information. Research designs also differed: (1) two studies used a three-group comparison [e.g., theatre majors versus some-acting versus non-acting; acting versus dancing versus psychology students (Gentzler et al., 2020; Schmidt et al., 2021)]; (2) five used a two-group comparison (actor versus non-actor) (Krahmer and Swerts, 2008; Goldstein, 2011; Klinge et al., 2012; Jurgens et al., 2015; Höfling et al., 2022); (3) one used a pre-post training model (Sun and Okada, 2021), and (4) one focused solely on a single group of professional actors (Orzechowicz, 2008).

#### 3.3.2 Clinical context

Twelve articles applied acting training or intervention to help people with diverse clinical conditions, such as brain injury, fibromyalgia, foetal alcohol spectrum disorder (FASD), neurodevelopmental disorder, schizophrenia, autism spectrum, emotional difficulties, preschoolers with special needs, and a clinical risk for psychosis. Regarding the experimental design, eight studies exclusively employed a pre-versus-post design with a single group (i.e., patient group), while two studies (Agnihotri et al., 2014; Mele et al., 2019) utilised both pre-versus-post and patients-versus-control comparisons. Three studies relied on pre-versus-post but also recruited another patient group without intervention (Corbett et al., 2011; Tang et al., 2020; van Huisstede et al., 2024), (see Table 1).

All the selected articles utilised acting interventions that involved various activities, such as improvisational acting, role-playing, rehearsals, and public performances. The acting interventions were led by at least one professional acting artist, and the intervention hours varied, ranging from 20 to 80 h. There was no preferred intervention duration, which also differed across studies, ranging from 3 weeks to 3 months. Notably, two studies did not provide information about the duration of the intervention.

#### 3.3.3 Occupational training

Five studies implemented acting training with participants in specific occupational contexts. The contexts include nursing students or students in teaching degrees, air force students, and clinic nurses. Considering the experimental design, three of the research studies employed a pre-versus-post-intervention approach; one utilised a control-versus-experimental group design (Albal et al., 2021), and another applied a control-versus-experimental-versus-placebo group design (Sisman and Buzlu, 2022).

The interventions in these five studies consisted exclusively of acting training. The interventions included psychodrama (Albal et al., 2021), creative drama and forum theatre (Briones et al., 2022), non-verbal and verbal communication workshops (Del Vecchio et al., 2022), an emotion-focused training program (Sisman and Buzlu, 2022), and theatre-based leadership development programs (Firing et al., 2022). Regarding the duration and frequency of the intervention, among the five research studies, one was conducted as a single session lasting 30 to 90 min, three lasted eight to 10 weeks, and the other lasted for one semester (approximately 3 months) as part of professional courses.

#### 3.3.4 Education and development

Six articles utilised acting training to support young people’s development. Three of these articles recruited participants from schools, but one of them did not specify the recruitment method. While most did not report pre-existing physical or mental health conditions, one study involved participants from underprivileged immigrant neighbourhoods, including people affected by war and undiagnosed neurodevelopmental conditions (Rousseau et al., 2012), and another examined Syrian refugees (Gürle, 2018). All studies compared participants’ pre-training performance with their post-training performance, and two of these studies also had either a non-intervention control group (Rousseau et al., 2012) or an active control group (Celume et al., 2020).

All selected articles utilised acting interventions that involved various acting intervention activities. Most articles had fewer and shorter sessions (i.e., four to six sessions and 60–70 min for each session), except for two that had longer sessions (12 90-min sessions and five 120-min sessions), compared with other application areas. This might be because the participants were young children who may be less patient or more easily distracted.

### 3.4 The effects of acting training

#### 3.4.1 Emotion recognition

Both quantitative and qualitative evidence from the included studies revealed significant improvements in participants’ ability to recognise and understand their emotions and those of others. For example, Klinge et al. (2012) found that professional actors’ auditory emotion discrimination accuracy was comparable to that of blind individuals, who rely heavily on auditory cues, and superior to that of sighted controls. Similar effects were observed among professionals such as nurses and teachers, whose perceived emotional awareness and understanding of their own and others’ emotions improved following acting-based interventions (Albal et al., 2021; Briones et al., 2022; Del Vecchio et al., 2022; Sisman and Buzlu, 2022). Clinical studies also reported comparable benefits: inpatients with schizophrenia spectrum disorder who participated in theatre activities showed higher facial emotion recognition accuracy, particularly for fear and sadness, than non-participants (Mele et al., 2019). Moreover, a child with a neurodevelopmental disorder demonstrated improved understanding of others’ emotions after a performative theatre intervention (Eschenauer et al., 2023). Other studies noted increased emotional awareness among participants with autistic traits or Foetal Alcohol Spectrum Disorder (Keightley et al., 2018; Pires et al., 2020; Robinson and Kalawski, 2022).

However, some studies, particularly those with a clinical population, reported only non-significant trends towards improvement. Research involving children with autism spectrum disorder, adolescents with acquired brain injury, or young people at clinical risk for psychosis found positive but non-significant changes in facial emotion recognition (Corbett et al., 2011; Agnihotri et al., 2012, 2014; Tang et al., 2020), possibly due to small sample sizes or challenging delivery among clinical populations compared to non-clinical populations.

These findings suggest a complex and context-dependent influence of acting interventions on emotion recognition. Longitudinal evidence indicated that acting training may shape social-cognitive development in distinctive ways: after a year of training, acting students showed weaker correlations between Theory of Mind and empathy compared to peers in other arts disciplines, suggesting that acting fosters specialised and differentiated emotional skills (Goldstein, 2011). Furthermore, although professional actors and blind individuals (i.e., individuals with naturalistic learning of audio emotion recognition) performed similarly in auditory emotion discrimination, their neural activation patterns differed. Actors showed increased anterior cingulate and decreased amygdala activation compared with blind group, implying distinct underlying mechanisms (Klinge et al., 2012). Taken together, these findings indicate that acting-based interventions can enhance emotional knowledge and recognition skills through deliberate, embodied, and reflective practice, which is distinct from the naturalistic learning of emotion recognition observed in everyday social interactions.

#### 3.4.2 Emotion expression

Evidence from the included studies indicated that acting interventions could enhance individuals’ emotional expression by increasing both the diversity and intensity of expressive behaviours. For example, drama-based interventions for young children led to improvements in motor skills and more embodied emotional behaviours, namely combining gesture, facial, and vocal expressions, when recalling story characters’ emotions (Nikiforidou and Stack, 2019; van Huisstede et al., 2024). Similarly, undergraduate students participating in a semester-long physical theatre course self-reported feeling “freer” in their emotional and bodily expression, suggesting a release from previous inhibitions (Kosma et al., 2024). Across both children and adults, including nurses and participants with clinical conditions, theatre-based interventions improved functional expressive communication, reflected in enhanced verbalisation of emotions, clarity of expression, and self- or observer-rated expressive abilities (Agnihotri et al., 2014; Keightley et al., 2018; Albal et al., 2021; Sisman and Buzlu, 2022).

Experimental comparisons between actors and non-actors revealed mixed effects. Höfling et al. (2022) used Automatic Facial Coding and found that actors’ posed expressions were rated as significantly more intense for joy, anger, surprise, sadness, disgust, and fear, with greater facial muscle activation for joy and anger. Likewise, Jurgens et al. (2015) reported a broader range of vocal expressiveness among actors compared to non-actors. However, the same study found no difference in listeners’ emotion recognition accuracy between the two groups, and sadness was recognised more accurately in spontaneous, authentic speech than in acted portrayals. Similarly, Krahmer and Swerts (2008) observed that while intensity differed between professional and novice actors and authentic expressions, perceived authenticity did not.

Horwitz et al. (2010) offered further insight into context-dependent effects of expression. In their study, patients with fibromyalgia rated their own video-recorded performances as significantly more emotionally intense when acting alongside a professional actor compared to performing alone or expressing emotion through dance. This suggests that the social and interactive context of acting can amplify emotional expression.

Taken together, these findings indicate that acting-based interventions enhance expressive range and intensity, yet the perceived authenticity of acted emotions may depend on context and interpersonal engagement. Performed emotions may therefore appear more intense, but not necessarily more genuine, than naturally occurring expressions.

#### 3.4.3 Other skill development

Beyond emotion recognition and expression, acting-based interventions were associated with the development of a broad and interrelated set of competencies, underscoring the pluridimensional nature of skills fostered through arts-based practice. Across studies, outcomes extended beyond emotional literacy to include cognitive and metacognitive abilities, social and interpersonal skills, self-management capacities, and embodied and creative process skills.

At the cognitive level, acting-based activities supported perspective-taking, self-reflection, imagination, and executive functioning. Role-play and improvisation require participants to infer others’ mental states and adopt alternative viewpoints, thereby strengthening theory of mind and reflective self-awareness (Agnihotri et al., 2012; Briones et al., 2022; Eschenauer et al., 2023). Several studies also reported improvements in executive functions such as inhibition, cognitive flexibility, concentration, and emotional control, reflecting the structured yet adaptive cognitive demands of rehearsal and performance contexts (Agnihotri et al., 2014; Eschenauer et al., 2023).

In parallel, interventions consistently enhanced social and interpersonal functioning. Participants demonstrated enhanced social and communication skills, including better turn-taking, topic maintenance, and teamwork (Agnihotri et al., 2014; Del Vecchio et al., 2022; Firing et al., 2022). Qualitative evidence also highlighted perceived gains in social interaction and cohesion, social integration, and relationship-building among diverse populations, suggesting that the collective and relational nature of acting-based work provides a supportive context for practising social interaction (Keightley et al., 2018; Firing et al., 2022).

Acting-based interventions were also associated with gains in self-management and regulatory capacities. Beyond recognising or expressing emotions, participants reported increased self-confidence, self-efficacy, coping skills, and resilience, alongside reductions in distress or clinical symptoms in some populations (Horwitz et al., 2010; Agnihotri et al., 2012; Tang et al., 2020). These outcomes point to the development of more advanced regulatory skills, including the ability to modulate emotional responses, manage internal states, and respond adaptively to challenging situations (Gentzler et al., 2020).

Finally, several studies emphasised embodied and creative process skills as distinctive features of acting-based interventions. Movement, posture, and sensory awareness were central to learning processes, supporting body awareness, embodied cognition, and creative engagement (Horwitz et al., 2010; Eschenauer et al., 2023). Participants frequently described heightened bodily awareness, spontaneity, and immersive engagement in creative tasks, suggesting that learning occurred through the integration of physical, cognitive, and emotional processes.

Together, these findings indicate that acting-based interventions engage a broad constellation of skills that extend beyond emotional processing, underscoring their potential to support integrated cognitive, social, regulatory, and embodied processes.

## 4 Discussion

This review, which aimed to examine the impact of acting training on emotion recognition and expression, found evidence that such training can enhance these skills in individuals. Although the included articles employed a wide range of acting methods and research designs and demonstrated varied levels of methodological quality, which may weaken the overall strength of evidence, this review nevertheless provides the first overview of existing research in this area. As no prior systematic review has been conducted, it offers an initial exploration that may help illuminate the mechanisms underlying acting-based interventions and guide future research directions. The selected articles indicated that acting intervention could demonstrate similar improvement in emotion recognition skills comparable to those achieved through naturalistic learning and enhance one’s ability to be aware of their own and others’ emotions. However, some studies, particularly with clinical populations, reported challenges in obtaining statistically significant evidence for improvements in emotion recognition skills. In terms of emotion expression, the selected articles also showed that acting-based intervention could broaden and intensify expression behaviours, increasing variety, intensity, and frequency of emotion expression. Nonetheless, several studies pointed out that such intervention did not necessarily enhance the authenticity of the acted emotion expression when compared with naturally occurring expression.

In the following section, we discuss six key areas: challenges in assessing emotion recognition and expression, intervention design, underlying mechanisms, individual and population differences, practical implications, and limitations.

### 4.1 Challenges in assessing emotion recognition and expression

We found that quantitative research tools for emotion recognition primarily focused on facial cues, overlooking other important channels of emotional communication. This narrow focus may not fully capture the impact of acting training on overall emotion recognition skills. Human emotional communication occurs through multiple modalities, including face, voice and bodily movement (Van den Stock et al., 2008; de Gelder et al., 2015). The ability to recognise facial expressions does not necessarily equate to recognising emotions conveyed through other forms of expression, as studies have shown that the recognition of emotions from faces can differ from that based on body movements (van de Riet and de Gelder, 2008). Acting training, in particular, emphasises the embodied understanding of emotion (Nikiforidou and Stack, 2019; van Huisstede et al., 2024), fostering awareness that extends beyond facial cues alone. Therefore, assessment tools for emotion recognition should incorporate additional modes of human expression, such as bodily emotion recognition tasks like “The Bodily Expressive Action Stimulus Test” (De Gelder and Van den Stock, 2011). Developing instruments that integrate vocal and physical cues could help overcome the limitations of existing measures and better capture the full spectrum of emotional signals encountered in real-world interactions.

Regarding emotion expression, the selected studies rely predominantly on qualitative methods, revealing a notable lack of quantitative tools capable of effectively measuring improvements in expressive abilities. We recommend the development of new assessment instruments, such as the deep machine learning tool Automatic facial coding software (Höfling et al., 2022) or adapting existing assessment frameworks (Chou et al., 2017). For example, in Chou et al. (2017), actors recorded emotion expressions that were subsequently evaluated for accuracy by themselves, peer directors and non-acting-experience observers. The averaged ratings across these evaluators provided a more objective measure of expressive accuracy, offering a valuable model for future research.

Acting training offers high environmental validity, as it simulates real-world interactions and support the transfer of skills to everyday life (Macneill et al., 2016). However, most of the studies included in this review still rely on assessment tools conducted in controlled environments or use only single-source emotional stimuli, such as static facial or bodily expressions. Such discrepancy between the training and assessment may not fully capture the effectiveness of acting training in authentic emotional communication. Future research should adopt assessment methods that more closely reflect real-world interactions, such as analysing complex emotions conveyed through vocal tone, body movement, and contextual performance in dynamic video scenarios. Alternatively, qualitative field observations or the arrangement of real social interaction scenarios may be employed, allowing observers or interaction partners, as external evaluators, to assess differences in participants’ emotional recognition and expression abilities. This will ensure the alignment of environmental validity between the training and assessments.

### 4.2 Acting training design

This review identified two main issues contributing to the complexity of acting-based interventions: the inclusion of multiple training components and insufficient detail on training procedures. For example, Agnihotri et al. (2012) listed ten practices in their theatre programme, such as voice work, movement, mask work, and clowning, delivered over 4 h daily for 3 weeks, without specifying the duration or frequency of each component or describing the content of individual exercises. Similarly, while several studies [e.g., (Agnihotri et al., 2012; Keightley et al., 2018)] incorporated voice work, their emphases varied from vocal authenticity to expressiveness, leading to inconsistent outcomes. Although positive effects on emotion recognition and expression were generally reported, the lack of standardisation limits the replicability and refinement of interventions in future research. This multi-component nature resembles challenges commonly encountered in complex behavioural interventions such as Cognitive Behavioural Therapy, which comprises a family of related approaches (e.g., cognitive therapy, dialectical behaviour therapy, acceptance and commitment therapy, mindfulness-based cognitive therapy). These approaches integrate cognitive restructuring, behavioural rehearsal, mindfulness, and emotion-regulation strategies, making it difficult to isolate which components drive change (McMain et al., 2015).

### 4.3 Underlying mechanisms of acting interventions on emotion skills

The findings of this review suggest that the benefits of acting training are not derived from mere repetition but from a goal-directed simulation of an agent’s inner state. This process appears to be driven by top-down, reflective mechanisms rather than by modifying bottom-up emotional reactivity. Based on the evidence, we propose a working model in which improvements are driven by several mutually reinforcing processes.

First, training involves conceptual enrichment, expanding participants’ explicit emotional knowledge. Through script analysis, character work, and facilitator feedback, participants learn to differentiate nuanced emotional states (e.g., anger vs. contempt) and their corresponding appraisals. This enriched conceptual repertoire, as seen in the improved awareness reported by nurses and teachers (Albal et al., 2021; Briones et al., 2022; Del Vecchio et al., 2022; Sisman and Buzlu, 2022), likely supports finer-grained recognition and more precise expression.

Second, rehearsal fosters perceptual-motor tuning, or embodied mapping. Participants iteratively link internal states to coordinated facial, vocal, and postural patterns. This embodied practice, which aligns with findings of improved motor skills and embodied emotional behaviours (Nikiforidou and Stack, 2019; van Huisstede et al., 2024), calibrates the intensity, timing, and clarity of expressive signals.

Third, acting requires significant executive control and reappraisal. Performers must inhibit their own spontaneous feelings to convincingly portray a character’s objectives. This aligns with the neural evidence from Klinge et al. (2012), a key study in our review. They found that professional actors, when processing angry prosody, exhibited down-regulation of the amygdala mediated by the Anterior Cingular Cortex. This suggests a highly developed, top-down cognitive control strategy that supports rapid emotion classification without being captured by a reactive bottom-up emotional response.

Thus, these mechanisms provide a rationale for improvements in both emotional skills. Gains in expression are intuitively driven by conceptual enrichment (providing precision) and perceptual-motor tuning (providing calibrated intensity and clarity). While the pathway from acting to improved expression is intuitive, this model also provides a clear theoretical rationale for the observed gains in emotion recognition. We propose this occurs via three routes: (1) Category learning, where explicit coaching sharpens the boundaries between emotions, aiding discriminability; (2) Production-perception coupling, whereby practicing the production of nuanced vocal and facial cues builds the internal models needed to decode them in others; and (3) Top-down control, as demonstrated by Klinge et al. (2012), which allows observers to focus on diagnostic emotional cues while inhibiting misleading information.

Finally, this model accounts for the mixed findings on authenticity. Several studies found that acted expressions were more intense but not necessarily more authentic or accurately recognised (Krahmer and Swerts, 2008; Jurgens et al., 2015). This reflects a trade-off: acting training prioritises signal clarity and legibility for an audience. This may result in expressions that are highly intense (Höfling et al., 2022) but lack the subtle idiosyncrasies of spontaneous, naturally occurring affect. Understanding these distinct mechanisms is crucial for tailoring future interventions.

It is important to note, however, that these proposed mechanisms must be interpreted with caution. As identified in our review, several of the included studies were limited by small sample sizes or the absence of active control groups. Therefore, while this framework provides a strong conceptual starting point supported by preliminary evidence, it requires rigorous validation through future studies with larger, more diverse samples and robust controlled designs. Nonetheless, this synthesis provides an essential initial step, illuminating potential pathways and establishing acting-based interventions as a promising and valuable area for future psychological inquiry.

### 4.4 Individual and population factors influencing outcomes

While most findings consistently indicate that acting training enhances emotion recognition and expression, some inconsistencies emerged regarding its effects on specific emotions [e.g., (Jurgens et al., 2015; Gentzler et al., 2020)]. These variations may partly reflect differences in participant populations. Many interventions targeted clinical groups, such as individuals with acquired brain injury or autism spectrum disorder, who often present with baseline difficulties in emotion processing and Theory of Mind (ToM). This is particularly relevant as several studies employed measures like the Reading the Mind in the Eyes Test (RMET), originally developed to detect subtle ToM impairments in autism (Baron-Cohen et al., 2001). However, such samples introduce additional complexity. As McDonald et al. (2013) argued, even when interventions successfully improve emotional skills, co-occurring cognitive deficits in attention or executive function may constrain observable performance gains, producing non-significant outcomes. This may account for inconsistent findings for emotions such as sadness. For example, Agnihotri et al. (2014) reported that theatre training improved sadness recognition among participants with acquired brain injury, whereas studies with non-clinical student samples found no such effect (Goldstein, 2011; Gentzler et al., 2020). In these latter cases, null effects may reflect higher baseline emotional competence or ceiling effects typical of non-clinical populations (Calvo et al., 2014; Israelashvili and Fischer, 2022). However, most selected studies did not control for individual differences such as autistic traits, prior emotional experience, or cognitive functioning, which could influence both baseline ability and responsiveness to training. Future research should account for these factors to better isolate the specific effects of acting interventions.

### 4.5 Implications for the study of emotional intelligence and related constructs

The initial research objective of this study was to observe whether acting training enhances the ability to recognise and express emotions. Our results indicate that alongside these outcomes, various studies also explored abilities beyond emotional recognition and expression, which may overlap with emotional intelligence (EI) skills.

EI was first introduced by Salovey and Mayer (1990), who defined it as “the ability to monitor one’s own and others’ feelings and emotions, to discriminate among them and to use this information to guide one’s thinking and actions” (p. 189). According to Salovey and Sluyter's (1997) widely accepted model of EI, the four main branches of EI are: (1) Reflective Regulation of Emotions to Promote Emotional and Intellectual Growth; (2) Understanding and Analysing Emotions; Employing Emotional Knowledge; (3) Emotional Facilitation of Thinking; (4) Perception, Appraisal, and Expression of Emotion (p. 11).

Based on the aforementioned definition of EI and the findings of this study, acting training may also contribute to an overall improvement in EI. Verducci (2000) already found that dramatic arts can be a tool to teach emotional intelligence. For instance, research has found that two crucial abilities for better social interaction are emotion recognition (Martinez et al., 2016; Kaltwasser et al., 2017; Israelashvili and Fischer, 2022) and expression (Tracy et al., 2015; Chervonsky and Hunt, 2017). Moreover, researchers have found that acting training can improve people’s capacity to face daily challenges (Levy, 1997; Emunah, 2019) and enhance emotional awareness and social communication skills (Cameron, 1999). Acting can be labelled a natural and essential life skill that most people are familiar with (Milroy, 1982). Therefore, we suggest that future studies on the impact of acting training in psychology research should consider expanding its scope to include the entire emotional intelligence spectrum. This could provide more effective training tools for psychology and deepen the connection between drama and psychology.

### 4.6 Limitations

#### 4.6.1 The current state of literature

Our review indicates several limitations and constraints of the current literature: exclusion of needed information in the published paper, lack of comparison groups, and not controlling for confounding factors. First, some of the included studies did not have the necessary details (e.g., participant information, training contents or assessment tools), which restricts the comparison across studies and future research replication. Precise details of research design are crucial for enhancing replicability and guiding future research endeavours. Nevertheless, some included studies failed to provide the comprehensive information necessary for replication, such as participants’ features and intervention details. We recommend that future research utilises guidelines, such as the CONSORT checklist, which includes extension versions (Zwarenstein et al., 2008), to ensure robust and transparent reporting practices. The other limitation was in the research design of these selected articles. Some studies [e.g., (Agnihotri et al., 2012)] did not include a control group for comparison, which makes it hard to support the idea that acting training could improve one’s emotional skills. Furthermore, long intervention durations might simultaneously include numerous uncontrolled research variables during the intervention period. For example, personal life experiences at home (Pollak and Kistler, 2002) or school (Mudge et al., 2006) could alter their ability to express or recognise emotions. Also, although some studies include control groups, they did not control for individuals’ previous life experiences, which could be a confounder of changes in emotion recognition and expression skills. Additionally, only Agnihotri et al. (2012) and Sisman and Buzlu (2022) conducted long-term post-tests, with durations of 8 months and 6 months, respectively. It is unclear whether the positive effects observed can be sustained in the participants’ future lives. Future research could conduct longitudinal studies to evaluate the enduring impact of the interventions.

#### 4.6.2 Limitations of the current study

In this review, we were only able to include papers written in English or Chinese, but some publications in other languages could not be identified or selected. This may have limited the number of articles included. Moreover, this review did not include other performing arts such as singing or dance, yet they have also been shown to enhance emotional skills [e.g., (Martins et al., 2021)]. This focus was chosen due to the unique multimodal nature of acting, its close resemblance to real-life emotional communication, the considerable variation in acting methods, and the lack of existing systematic reviews in this area. Future work should compare acting with other performing arts to build a more comprehensive understanding of how different art forms contribute to emotional development. We also found that several studies included in this review were of relatively low methodological quality according to the MMAT assessment, with some not fully meeting the appraisal criteria. This limitation affects the overall reliability and validity of the review’s conclusions. Nonetheless, these studies offer valuable exploratory insights, highlighting emerging directions and overlooked gaps that can inform more rigorous and comprehensive investigations in future research. Another limitation of this review is the inclusion criterion that required all interventions to be delivered by trained drama professionals. Therefore, future research could adopt a broader definition of performance-based interventions, encompassing activities such as role-play or drama therapy, to compare the effectiveness of professionally led versus researcher- or teacher-led interventions. A further consideration relates to how methodological quality appraisals are interpreted in the context of psychosocial and acting-based interventions. In this review, the MMAT was used to assess methodological rigour and to identify areas where future studies could strengthen design, measurement, and evaluability. Lower ratings on specific criteria do not imply that studies lacked meaningful or practice-relevant insights but rather highlight challenges in operationalising and empirically capturing complex, context-sensitive processes. Emotional skills targeted in acting-based interventions are embedded within broader psychosocial competencies that integrate cognitive, emotional, and social processes. As emphasised by Durlak et al. (2011), these domains are interdependent and unfold dynamically within specific settings. Future research would benefit from more rigorous and transparent methodological approaches that preserve this complexity while employing clearer evaluative frameworks, thereby enhancing both the interpretability and impact of findings.

## 5 Conclusion

This systematic review examined the empirical applications of acting training and its effects on emotional skills. The findings support the notion that acting training can enhance individuals’ emotion recognition and expression abilities. Moreover, existing studies have demonstrated the use of acting training beyond the arts, extending into clinical, educational, and professional development contexts. However, the considerable heterogeneity in study design, training content, and outcome measures limits the comparability and generalisability of findings across studies. Despite these variations, our review offers recommendations for improving research design and reporting practices to strengthen replicability and methodological rigour. In addition, we propose a working model illustrating how acting training may influence emotional development. This is particularly relevant given that acting training appears to foster key emotional and socio-emotional skills crucial to individuals’ wellbeing and success. Future research should further investigate specific components of emotional functioning to disentangle the mechanisms underlying these effects and explore how acting-based approaches can be effectively applied beyond clinical settings to maximise their broader impact.

## Data Availability

The raw data supporting the conclusions of this article will be made available by the authors, without undue reservation.
