# Supplementary material for: The impact of acting training on emotion recognition and expression: a systematic review
Source: Front Psychol. 2026 Feb 16;17:1749252. doi: 10.3389/fpsyg.2026.1749252 (PMC12950610; doi:10.3389/fpsyg.2026.1749252)
Supplement: Supplementary file 1 [file Table_1.DOCX]

Supplementary Material

# Supplementary Table 1. Research tools

| **Emotion "recognition" measures.** | | | |
| --- | --- | --- | --- |
| **Quantitative** | **Measure** | **Reference** | **Applied papers** |
|  | A computerized emotion discrimination task | -- | Agnihotri et al. (2014) |
|  | Adapted paper version (Monnier et al., 2018) of the valence and arousal Self-Assessment Manikin scale (SAM)** | Bradley and Lang (1994) | Celume and Zenasni (2022) |
|  | Amsterdam Dynamic Facial Expression Set | van der Schalk et al. (2011) | Gentzler et al. (2020) |
|  | BarOn Emotional Quotient Inventory: Youth Version Short Form (EQ-i:YV) | Bar-On and Parker (2000) | Agnihotri et al. (2012) |
|  | Basic Empathy Scale for Adolescents | Jolliffe and Farrington (2006) | Goldstein (2011) |
|  | Canadian Occupational Performance Measure (COPM) | -- | Agnihotri et al. (2012) |
|  | Empathic Accuracy Paradigm | Ickes (2001) | Goldstein (2011) |
|  | Empathy Quotient (EQ) | Baron-Cohen and Wheelwright (2004) | Schmidt et al. (2021) |
|  | Interpersonal Reactivity Index (IRI) | Davis (1980) | Schmidt et al. (2021) |
|  | Levels of Emotional Awareness Scale* | Kuzucu (2008) | Sisman and Buzlu (2022) |
|  | Levels of Emotional Awareness Scale (LEAS), adapted for Turkish | Kuzucu (2008) | Albal et al. (2021) |
|  | Penn computerized neurocognitive battery, CNB | Moore et al. (2015) | Tang et al. (2020) |
|  | Reading the Mind in the Eyes test | Baron-Cohen et al. (2001) | Goldstein (2011); Schmidt et al. (2021) |
|  | Reading the Mind in the Eyes Test, Child Version (RMET-G), Self-adapted French version * | Baron-Cohen et al. (1997) | Celume et al. (2020); Celume and Zenasni (2022) |
|  | Self-developed 5-point Likert scale paper surveys | -- | Del Vecchio et al. (2022) |
|  | Self-report on cognitive and affective empathy | López-Pérez et al. (2008) | Briones et al. (2022) |
|  | NEPSY Affect Recognition | Korkman et al. (2007) | Corbett et al. (2011) |
|  | Emotion discrimination task | -- | Klinge et al. (2012) |
|  | FMRI measuring amygdala activation | -- | Klinge et al. (2012) |
| **Qualitative** | **Measures** | **Reference** | **Applied papers** |
|  | Field study | -- | Orzechowicz (2008); Rousseau et al. (2012); Sun and Okada (2021); Firing et al. (2022) |
|  | Focused group | -- | Agnihotri et al. (2012); Keightley et al. (2018); Tang et al. (2020) |
|  | Interview | -- | Orzechowicz (2008); Rousseau et al. (2012); Sun and Okada (2021); Firing et al. (2022) |
|  | Self-developed paper surveys | -- | Del Vecchio et al. (2022) |
| **Quantising qualitative** | **Measures** | **Reference** | **Applied papers** |
|  | Framework Analytic Approach | Ritchie and Spencer (2002) | Agnihotri et al. (2012) |
|  | Observational instrument of motor skills (OSMOS)  Cognitive Emotional Skills: 6-item version of LEAS-C*** | Castañer et al. (2009)  Bajgar et al. (2005) | Nikiforidou and Stack (2019)  Eschenauer et al. (2023) |
| **Emotion "expression" measures** | | | |
| **Quantitative** | **Measures** | **Reference** | **Applied papers** |
|  | Communication Skills Inventory (CSI) | Ersanlı and Balcı (1998) | Albal et al. (2021) |
|  | Demographic characteristics questionnaire. | -- | Albal et al. (2021) |
|  | Emotional Expression Scale | Kuzucu (2011) | Sisman and Buzlu (2022) |
|  | Emotion discrimination task | Klinge et al. (2010) | Klinge et al. (2012) |
|  | FaceReader software (FR; Version 7.1, Noldus Information Technology) and aggregated with Observer XT offline (Version 12.5,Noldus Information Technology). | -- | Höfling et al. (2022) |
|  | Self developed 5-point Likert scale paper surveys | -- | Del Vecchio et al. (2022) |
|  | the program NBS Presentation (Neurobehavioral Systems, Inc., Albany, California) | -- | Jurgens et al. (2015) |
|  | Video interpretation: evaluation of emotional expression | -- | Horwitz et al. (2010) |
|  | Self-report emotion questionnaire* | Mackie and Worth (1989) | Krahmer and Swerts (2008) |
|  | Observer ratings of participants’ expressions (7-point valence scale) | -- | Krahmer and Swerts (2008) |
|  | Story Recall Measure (SRM) | Petersen and Spencer (2016) | van Huisstede et al. (2024) |
| **Qualitative** | **Measure** | **Reference** | **Applied papers** |
|  | Clinical Change Interview (CCI) | Elliott et al. (2001) | Pires et al. (2020) |
|  | Client-Emotional Processing Scale for Autism Spectrum | Robinson and Elliott (2016) | Robinson and Kalawski (2022) |
|  | Field study | -- | Orzechowicz (2008); Rousseau et al. (2012); Nikiforidou and Stack (2019); Sun and Okada (2021); Celume and Zenasni (2022); Firing et al. (2022) |
|  | Focused group | -- | Agnihotri et al. (2012); Keightley et al. (2018); Tang et al. (2020) |
|  | Interview | -- | Rousseau et al. (2012); Pires et al. (2020); Sun and Okada (2021); Firing et al. (2022); Robinson and Kalawski (2022); Kosma et al. (2024) |
|  | Self-developed paper surveys | -- | Del Vecchio et al. (2022) |
|  | Exploratory unstructured observation | -- | Gürle (2018) |
| **Quantising qualitative** | **Measure** | **Reference** | **Applied papers** |
|  | Framework Analytic Approach | Ritchie and Spencer (2002) | Agnihotri et al. (2012) |
|  | Helpful Aspects of Therapy (HAT) | Llewelyn et al. (1988); Sales et al. (2007) | Pires et al. (2020) |
|  | Observational instrument of motor skills (OSMOS)  Structured behavioural observation | Castañer et al. (2009)  -- | Nikiforidou and Stack (2019)  Abdulhaq et al. (2025) |
|  | Observation using the Embodiment Coding System (ECS) | Bernstein et al. (2024) | van Huisstede et al. (2024) |
| * Means the measure was translated to different languages.  ** Means the tool had been adapted to meet the researcher’s need. | | | |

# Supplementary Table 2. Other measures (not emotion recognition and expression)

| **Social skills (including interaction, communication, collaboration, etc.)** | | | |
| --- | --- | --- | --- |
| **Topics** | **Measures** | **Reference** | **Applied papers** |
| Awareness of professional conflicts (intrapersonal and interpersonal) | Cross-sectional, qualitative design | Gibbs (2012); Glaser and Strauss (2017) | Briones et al. (2022) |
| Children’s daily living competency | Adaptive behavior assessment system second edition parent form (ABAS:II) | Harrison and Oakland (2000b) | Agnihotri et al. (2012) |
| Collaborative | Prisoner’s dilemma (PD) task | Landazabal (1995) | Celume et al. (2020) |
| Communication | Profile of pragmatic impairments in communication (PPIC) | Linscott et al. (1996) | Agnihotri et al. (2014) |
|  | Communication skills inventory (CSI) | Ersanlı and Balcı (1998) | Albal et al. (2021) |
| Relational and social knowledge. | Interviews | Charmaz (2006) | Firing et al. (2022) |
| Social skills | Emotional quotient inventory: youth version short form (EQ-i:YV) | Bar-On and Parker (2000) | Agnihotri et al. (2012) |
|  | Child and adolescent scale of participation (CASP) | Bedell and Dumas (2004) | Agnihotri et al. (2014) |
|  | Goal attainment scaling (GAS) | Malec (1999) | Agnihotri et al. (2014) |
|  | paper surveys. | Self-developed | Del Vecchio et al. (2022) |
|  | social networks inventory | Treadwell et al. (1993) | Agnihotri et al. (2014) |
| Social desirability | Social Desirability Scale | Crowne and Marlowe (1960) | Klinge et al. (2012) |
| **Physical/mental well-being** | | | |
| **Topics** | **Measures** | **Reference** | **Applied papers** |
| Behaviour of the listener | R: A Language and environment for statistical computing. | R Team Core, 2019 | Jurgens et al. (2015) |
| Beliefs about controllability of emotions. | Implicit theories of emotion questionnaire. | Tamir et al. (2007) | Gentzler et al. (2020) |
| Embodied cognition | Observational instrument  of motor skills (OSMOS) | Castañer et al. (2009) | Nikiforidou and Stack (2019) |
| Emotional and behavioural difficulties | Strength and difficulty questionnaire (SDQ) | Goodman et al. (2008) | Rousseau et al. (2012) |
|  | The Conners comprehensive behaviour rating scale | Conners (1973) | Abdulhaq et al. (2025) |
| Emotion regulation | Emotion amplification and reduction scales (TEARS) | Hamilton et al. (2009) | Gentzler et al. (2020) |
|  | *Acceptance* and *focus and venting* subscales of the COPE. | -- | Goldstein (2011) |
|  | Cognitive reappraisal subscale of the emotion regulation questionnaire (ERQ) | Gross and John (2003) | Goldstein (2011); Gentzler et al. (2020) |
|  | Difficulties in emotion regulation scale (DERS) | Gratz and Roemer (2004) | Gentzler et al. (2020) |
|  | Cognitive Emotion Regulation Questionnaire | Garnefski and Kraaij (2006) | Klinge et al. (2012) |
|  | Field study | -- | Orzechowicz (2008) |
| Negative symptoms | Clinical assessment interview for negative symptoms (CAINS) | Kring et al. (2013) | Tang et al. (2020) |
| Positive and Negative symptoms | Positive and Negative Affect Schedule | Watson et al. (1988) | Klinge et al. (2012) |
| Pain and health level | Interview | -- | Horwitz et al. (2010) |
| Physical and mental perception | Interview | -- | Robinson and Kalawski (2022) |
|  | Intensity, pleasantness, threat, and speaker gender ratings | Klinge et al. (2010) | Klinge et al. (2012) |
| Psychosis symptoms | Structured interview for prodromal syndromes (SIPS); Corresponding scale of prodromal symptoms (SOPS); Global assessment of functioning (GAF) ratings | Miller et al. (2003) | Tang et al. (2020) |
| Anxiety | State-Trait Anxiety Inventory | Speilberger et al. (1970) | Klinge et al. (2012) |
| Quality of life | KID KINDL questionnaire | Ravens-Sieberer and Bullinger (1998) | Abdulhaq et al. (2025) |
| Self awareness | Interview | -- | Firing et al. (2022) |
| **Psychological abilities (e.g., empathy, self-esteem, leadership, creativity)** | | | |
| **Topics** | **Measures** | **Reference** | **Applied papers** |
| Affective empathy | Self-report | -- | Briones et al. (2022) |
| Creativity | Evaluation of creative potential (EPoC) | Lubart et al. (2011) | Celume and Zenasni (2022) |
| Leadership | Interviews | Charmaz (2006) | Firing et al. (2022) |
| Self-esteem | Rosenberg self-esteem scale | Rosenberg (1965) | Agnihotri et al. (2014) |
| **Effectiveness of specific topics (e.g., instructional resources, skill application, community activities, therapy, etc.)** | | | |
| **Topics** | **Measures** | **Reference** | **Applied papers** |
| Contribution of the TVPCT programme to their lives | A 22-item scale | Briones et al. (2015) | Briones et al. (2022) |
| Helpful aspects of Therapy | Helpful aspects of therapy (HAT) | Llewelyn et al. (1988); Sales et al. (2007) | Pires et al. (2020) |
| Occupational performance | Canadian occupational performance measure (COPM) | Law et al. (1994) | Agnihotri et al. (2012) |
| Participation enjoyment | Children’s assessment of participation enjoyment (CAPE) | Law et al. (2004) | Agnihotri et al. (2012) |
| Scale of participation | Child and adolescent scale of participation | Bedell (2004) | Agnihotri et al. (2014) |
| Usefulness of pedagogical resources of the TVPCT programme. | A cross-sectional and descriptive design used a 5-point Likert scale | -- | Briones et al. (2022) |
| **Others** | | | |
| **Topics** | **Measure** | **Reference** | **Applied papers** |
| Acoustic analysis  (Acoustic structure, speed, and pitch variability) | Linux MultiMedia Studio (LMMs)  <https://lmms.io/lsp/> | Bates (2010) | Jurgens et al. (2015) |
| Demographic characteristics | Questionnaire | -- | Albal et al. (2021) |
| Perceptions around language diversity and mastery of language | Interview | -- | Rousseau et al. (2012) |
| Promigratory experience | Interview | -- | Rousseau et al. (2012) |
| Socio-demographic | Interview | -- | Rousseau et al. (2012) |

# Supplementary Table 3. The difference in research intervention of drama acting training

| **Training duration** | **Counts** | **Applied papers** |
| --- | --- | --- |
| Short-term (within four weeks) | 4 | Gürle (2018); Nikiforidou and Stack (2019); Del Vecchio et al. (2022); Robinson and Kalawski (2022) |
| Medium-term (within eight weeks) | 5 | Horwitz et al. (2010); Agnihotri et al. (2012; 2014); Keightley et al. (2018); Celume et al. (2020) |
| Long-term (more than eight weeks) | 13 | Corbett et al. (2011); Rousseau et al. (2012); Pires et al. (2020); Tang et al. (2020); Albal et al. (2021); Sun and Okada (2021); Briones et al. (2022); Firing et al. (2022); Sisman and Buzlu (2022); Eschenauer et al. (2023); Kosma et al. (2024); van Huisstede et al. (2024); Abdulhaq et al. (2025) |
| No clear description of training duration | 1 | Celume and Zenasni (2022) |
| **Training frequency** | **Counts** | **Applied papers** |
| Single session | 1 | Robinson and Kalawski (2022) |
| Two to ten sessions | 6 | Gürle (2018); Nikiforidou and Stack (2019); Pires et al. (2020); Celume and Zenasni (2022); Del Vecchio et al. (2022); Eschenauer et al. (2023) |
| More than ten sessions | 16 | Horwitz et al. (2010); Corbett et al. (2011); Agnihotri et al. (2012); Rousseau et al. (2012); Agnihotri et al. (2014); Keightley et al. (2018); Celume et al. (2020); Tang et al. (2020); Albal et al. (2021); Sun and Okada (2021); Briones et al. (2022); Firing et al. (2022); Sisman and Buzlu (2022); Kosma et al. (2024); van Huisstede et al. (2024); Abdulhaq et al. (2025) |
| **Total training hours** | **Counts** | **Applied papers** |
| Less than 10 hours | 3 | Nikiforidou and Stack (2019); Celume and Zenasni (2022); Del Vecchio et al. (2022) |
| 10-20 hours (less than) | 6 | Rousseau et al. (2012); Gürle (2018); Celume et al. (2020); Pires et al. (2020); Sisman and Buzlu (2022); Eschenauer et al. (2023) |
| 20-30 hours (less than) | 1 | Horwitz et al. (2010) |
| 30 hours or more | 7 | Corbett et al. (2011); Agnihotri et al. (2012; 2014); Keightley et al. (2018); Tang et al. (2020); Albal et al. (2021); Kosma et al. (2024) |
| No clear indication of hours | 6 | Sun and Okada (2021); Briones et al. (2022); Firing et al. (2022); Robinson and Kalawski (2022); van Huisstede et al. (2024); Abdulhaq et al. (2025) |

# Supplementary Table 4. Drama acting training with detailed activities

| **Non-verbal / Physical training** | | | | |
| --- | --- | --- | --- | --- |
| **Topics** | **Count** | **Applied paper** | | **Notes** |
| Physical warm-up | 5 | Agnihotri et al. (2012); Rousseau et al. (2012); Agnihotri et al. (2014); Keightley et al. (2018)  Eschenauer et al., 2023; | |  |
| Three-dimensional awareness | 4 | Agnihotri et al. (2012; 2014); Keightley et al. (2018), Eschenauer et al., 2023; | |  |
| Voice work | 4 | Agnihotri et al. (2012); Rousseau et al. (2012); Agnihotri et al. (2014); Keightley et al. (2018) | |  |
| Breathing | 3 | Agnihotri et al. (2012; 2014); Keightley et al. (2018) | |  |
| Movement | 3 | Agnihotri et al. (2012; 2014); Keightley et al. (2018) | |  |
| Dramatization of a text with professional actors | 1 | Horwitz et al. (2010) | | Body training, acting training |
| Non-verbal communication | 1 | Del Vecchio et al. (2022) | | Empathetic Body Language, Mirroring, and Making Judicious Eye Contact |
| **Text/ Role/ Line Training** | | | | |
| **Topics** | **Count** | **Applied paper** | | **Notes** |
| Group dynamics | 5 | Agnihotri et al. (2012); Rousseau et al. (2012); Agnihotri et al. (2014); Keightley et al. (2018); Eschenauer et al. (2023) | |  |
| Story development | 5 | Agnihotri et al. (2012); Rousseau et al. (2012); Agnihotri et al. (2014); Keightley et al. (2018); Eschenauer et al. (2023) | |  |
| Character development | 3 | Agnihotri et al. (2012; 2014); Keightley et al. (2018) | |  |
| Script analysis | 2 | Agnihotri et al. (2012; 2014) | |  |
| Writing skills | 2 | Agnihotri et al. (2012; 2014) | |  |
| Dramatization of a text with professional actors | 1 | Horwitz et al. (2010) | | Reading text, vocal training, acting training |
| Story telling | 1 | Nikiforidou and Stack (2019) | |  |
| Verbal communication | 1 | Del Vecchio et al. (2022) | | Active listening, learning not to interrupt, and embracing silence |
| **Specific acting training method** | | | | |
| **Topics** | **Reference** | **Counts** | **Applied paper** | **Notes** |
| Clowning | Gray et al. (2012) | 3 | Agnihotri et al. (2012; 2014); Keightley et al. (2018) |  |
| Drama pedagogy training | Gioia et al. (2013); Merlino and Boal (2014) | 3 | Celume et al. (2020); Celume and Zenasni (2022); Abdulhaq et al. (2025) |  |
| Mask work | Rojas-Bermúdez and Moyano (2012); Rojas-Bermúdez (2017) | 3 | Agnihotri et al. (2012; 2014); Keightley et al. (2018) |  |
| Psychodrama | Rojas-Bermúdez and Moyano (2012); Özbek and Leutz (2013); Ü (2013); Rojas-Bermúdez (2017); Beauvais et al. (2019); Kaya and Deniz (2020) | 3 | Pires et al. (2020); Albal et al. (2021); Sisman and Buzlu (2022) | /Warm-up, enactment, and sharing/ diaphragmatic breathing exercises and body/sensorial  exploration. / mask role-playing of different life situations using the mask and dramatic games/ Techniques such as sociometry and doubling, role‐play, and mirroring |
| Spanish program “programa juego” | Landazabal (1995) | 2 | Celume et al. (2020); Celume and Zenasni (2022) | Warming up of feeling expression, face and body training |
| Alba emoting method (breathing and body awareness) | Bloch et al. (1987) | 1 | Robinson and Kalawski (2022) | Alba emoting method (breathing and body awareness) |
| Circus skills training | Gray et al. (2012) | 1 | Keightley et al. (2018) | Circus Skills Training |
| Collective sportive games (CSG) | -- | 1 | Celume et al. (2020) | Collective Sportive Games (CSG) |
| Early Years Educators at Play (EYEPlay) | Kilinc et al. (2016) | 1 | van Huisstede et al. (2024) |  |
| Image Theatre | Opdebeeck (2017) | 1 | Gürle (2018) | (Included improvisation, role play, puppetry, miming, masques  and character work.) |
| General drama training | -- | 1 | Firing et al. (2022) |  |
| Integrative art and drama therapy | -- | 1 | Abdulhaq et al. (2025) |  |
| Meisner technique | Meisner et al. (2012) | 1 | Sun and Okada (2021) | Repetition: involving moment-to-moment interaction in acting training |
| Physical Theatre | -- | 1 | Kosma et al. (2024) |  |
| Role play | -- | 1 | Nikiforidou and Stack (2019) | Role play |
| Social and emotional learning (SEL) approach | CASEL (2003) | 1 | Briones et al. (2022) | Creative drama and for UM theatre: |
| Theatre Improvisation Training to Promote Social Cognition (TIPS) | -- | 1 | Tang et al. (2020) | Theory, warm-up, performed, improvisation exercises, reflection |
| Theatre Musical acting, singing and dancing training | Social Emotional Neuro Science Endocrinology (SENSE): www.sensetheatre.com | 1 | Corbett et al. (2011) |  |

Note. Partial comprehensive drama acting training methods encompass both linguistic and non-linguistic aspects of performance. The statistics in this table will only be noted under the category of complete performance training methods, without duplicating statistics under the category of linguistic/non-linguistic performance training.

# Supplementary Table 5. Detailed information on the included papers by groups

| **Reference** | **Location** | **Age** | **Gender ratio** | **N** | **Participants Condition** | **Design** | **Training/treatment/intervention** | **Emotion expression/recognition measures/tasks** | | **Other measures/tasks** | **Emotion recognition and expression results** | **Other results** | |  |
| --- | --- | --- | --- | --- | --- | --- | --- | --- | --- | --- | --- | --- | --- | --- |
| **Actor/actress versus non-actor/actress** | | | | | | | | | | | | | |  |
| Gentzler et al. (2020) | USA(based on the first author's location） | M=19.86 | 70% F | 284 | Theatre major versus some-acting versus non-acting experience group | Group comparison | The study recruited the college students who were theatre majors, but did no mentioned specific training duration | Amsterdam Dynamic Facial Expression Set (van der Schalk et al., 2011); Cognitive reappraisal subscale of the emotion regulation questionnaire (ERQ, (Gross and John, 2003)) | | Experience with acting, Adult Temperament Questionnaire (ATQ) | Among the three groups, the theatre majors the most accurately identified pride expressions, no-acting group the most accurately identified anger expressions, and no significant differences in the recognition of other emotions (sadness, contempt, fear, disgust, embarrassment, joy, and surprise). | | Among the three groups, the theater majors reported: 1) higher temperamental sadness and fear; 2) more positive views about anger and sadness; 3) not more accepting of their own negative emotions (contrary to expectations); 4) more aware of their emotions, and 5) greater ability to amplify emotions but not necessarily better regulatory. | |
| Goldstein (2011) | USA(based on the first author's location） | 13-16 (M=14) | 67.92% F | 53 | Acting class versus  visual arts and music class | Group comparison | one-year acting or arts classes | Cognitive reappraisal subscale of the ERQ, (Gross and John, 2003); Empathic Accuracy Paradigm (Ickes, 2001); Basic empathy scale for adolescents (Jolliffe and Farrington, 2006); Reading the Mind in the Eyes test (RMET, (Baron‐Cohen et al., 2003) | | -- | No significant differences between the acting group and the other arts groups in their ability in the score of Fiction Emotion Matching, meaning no differences in recognition of sadness and scary. | | The adolescent actors seemed to separate their abilities in theory of mind from empathy and emotion regulation, while visual arts or music students showed correlations among these skills after one year of arts training. These findings suggest that different kinds of arts training may have different cognitive effects on social cognition. | |
| Höfling et al. (2022) | Germany | undergraduate | 100% F | 70 | Trained actress versus untrained | Group comparison | 70 female actors selected from three well-known picture inventories: The Karolinska Directed Emotional Faces, the Warsaw Set of Emotional Facial Expression pictures and the Radboud Faces Database | FaceReader software and aggregated with Observer XT offline (FR; Version 7.1, Noldus Information Technology; Version 12.5, Noldus Information Technology) | | -- | The trained actors produced more intense facial expressions compared to the untrained participants. | | The AFC measures revealed that the intensity of Action Unit activities was generally lower in pictures of untrained participants compared to actors’ pictures for all emotions. | |
| Jurgens et al. (2015) | Germany | 18-35 | Not mentioned | 288 | Actor versus non-actors | Group comparison | The study recruited professional actors through theatre agencies and drama schools, ensuring that they had received formal training and education. | NBS Presentation (Neurobehavioral Systems) was used to present the speech stimuli to the participants and collect their ratings of the specific vocal expression of emotion and authenticity. | | -- | Actors’ expressions were recognised more accurately than the non-actors’ expressions, but only for anger, fear, and joy stimuli, not for sadness, play-acted and authentic. | | Professional actors had more consistent articulation patterns than non-professional actors. | |
| Klinge et al. (2012) | Germany (University Medical Center Hamburg-Eppendorf) | M=35 | 50% F | 10 (actors) + previous data: 10 blind, 10 sighted | Professional actors (trained ≥ 3 years, ~4.75 hrs/week auditory/emotional speech training) versus blind versus sighted controls | Group comparison (fMRI experiment) | Extensive acting training focused on auditory emotional expression (intonation, timbre, vowel quality, intensity) with feedback | Emotion discrimination task (angry, happy, fearful, neutral prosody) (Klinge et al., 2010); Vowel discrimination task (control) (Klinge et al., 2010); fMRI measuring amygdala activation (LeDoux, 2007) | | Social Desirability Scale (Crowne and Marlowe, 1960); Cognitive Emotion Regulation Questionnaire (Garnefski and Kraaij, 2006); State-Trait Anxiety Inventory (Speilberger et al., 1970); Positive and Negative Affect Schedule (Watson et al., 1988); Intensity, pleasantness, threat, and speaker gender ratings (Klinge et al., 2010) Psycho-physiological interaction analysis of ACC–amygdala connectivity (Etkin et al., 2011) | The actors were significantly faster and more accurate than sighted controls, comparable to blind participants in emotion discrimination. However, the amygdala activation did not differ between actors and sighted and was significantly higher in blind participants. | | Actors rated emotional stimuli as less intense, particularly anger, than blind and sighted participants. ACC activation increased for angry stimuli, showing negative connectivity with amygdala (suggesting top-down regulation). Blind participants showed stronger amygdala responses and higher intensity ratings, indicating deprivation-induced plasticity rather than training effects. | |
| Krahmer and Swerts (2008) | The Netherlands | M = 27 / M = 36.2 | 59% F / 50% F | 70 / 40 | Experienced actors versus inexperienced actors versus non-actors | Group comparison | Actors were either from several theatre companies in Tilburg or were in their final year at Tilburg drama academy. All has between 3 and 25 years of professional experience (M = 11.2 years, SD = 6.5 years). | Participants had to rate the perceived emotional state from the actors’/non-actors’ stimuli on a 7-point valence scale in study 2. | | In study 1, emotion questionnaire derived from (Mackie and Worth, 1989). | Overall, all stimuli from participants in the data collection experiment (inexperienced actors, experienced actors and non-actors) accurately expressed a positive, negative or neutral emotion. However, surprisingly, expressions from professional actors were found to be more extreme and less realistic than non-professional actors. | | It was found that non-simulated expressions have a stronger impact on self-reported emotion scores than simulated expressions. Findings are consistent with the notion that actors no not feel the emotions they are acting out. | |
| Orzechowicz (2008) | USA(based on the first author's location） | -- | Not mentioned | -- | No comparison | Only actors | Actors were in acting classes and a theatre. | Over one thousand hours of fieldwork participant observation and seven semi-structured interviews were used with novice and semi-professional stage actors to gain insight into their experiences and strategies for managing emotions in the context of their work. | | -- | The study emphasised the structured nature of emotion management in the theatre and the resources that enable actors to effectively evoke specific emotions during their performances. | | The author argues that the theatre provides a space where feeling management strategies can be overtly developed, practiced, and discussed, and that actors are privileged emotion managers due to the resources and support available to them. | |
| Schmidt et al. (2021) | UK and abroad | undergraduate | 78.2% F | 176 | Acting versus  dancing versus  psychology students | Group comparison | The study focused on first-year graduate courses in acting, dance, or psychology. | RMET, (Baron‐Cohen et al., 2003); Empathy quotient (EQ, (Baron-Cohen and Wheelwright, 2004); Interpersonal Reactivity Index (IRI, (Davis, 1980) | | -- | Acting students showed the highest levels of emotion recognition among the three groups in, based on the results of RMET. | | Acting students reported above-average empathic concern, but did not experience strong distress. Dance students showed high levels of personal distress. Psychology students showed high levels of perspective-taking | |
| Sun and Okada (2021) | Japan | -- | Not mentioned | 26 | -- | Pre and post comparison | Activity training, Meisner technique, is a critical pair work based on Repetition to help participants learn how to interact with their partner in an imaginary situation. Activity training sessions, with a total of 82 sessions, each lasting about ten minutes. | The study used a qualitative analysis of the utterances made by actors during the training sessions to identify changes in their attentional focus and emotional expression over time. They also conducted interviews with two actors to gain insight into their subjective experiences of the training. | | The study analysed the characteristics of changes in actors during the acting training process by focusing on the pattern of utterances during the training. | By concentrating on their partners rather than themselves, actors were able to perform actions and responses in real time in specific situations, which led to real emotional involvement, rather than expressions. | | -- | |
| **Clinical context** | | | | |  |  |  | |  |  |  | |  | |
| Abdulhaq et al. (2025) | Jordan | 9-10 | 25% F | 12 | Behavioural and/or emotional difficulties (e.g. hyperactivity, anxiety, withdrawal, or peer issues). | Pre and post comparison | Ten weekly integrative visual arts therapy (drawing, painting and sculpting) and dramatherapy techniques (role-play, storytelling and movement-based games). The sessions were delivered by certified art therapists with additional training in drama-based therapeutic method with two trained observers to record behaviours The sessions were structured with an opening (breathing exercise), main activities and closing reflection. | Structured behavioural observations, including quantitative checklists on frequency of participation, instances of emotional expression, and quantitative notes on emotional breakthroughs or resistance. | | The Conners comprehensive behaviour rating scale (CBRS, (Conners, 1973), The KID KINDL questionnaire for measuring health-related QoL in children and adolescents (Ravens-Sieberer and Bullinger, 1998), and attendance | Noticeable transformation in increased children's participation, improved emotional articulation (confident use of body or facial expression), and better peer interactions. | | Significant improvements in child-reported QoL and teacher-rated hyperactivity, but no significant changes reported by parents. Authors argue the lack of objective emotion-recognition tasks and structured parental involvement | |
| Agnihotri et al. (2012) | Canada | 16&17 | 100% F | 2 | Severe childhood brain disorder | Pre and post comparison | The theatre skills training, including improvisation, vocal exercises, and role-playing, were designed to facilitate social skills and participation for youth living with childhood brain disorder. The training was led by three professional theatre artists and three master’s level occupational therapy students. The intervention lasted for three weeks, with daily sessions of four hours each. | Emotional discrimination task; BarOn Emotional Quotient Inventory (Bar-On and Parker, 2000); Canadian occupational performance measures (Law et al., 1994); Framework Analytic Approach (Ritchie and Spencer, 2002) | | Focus group; The children's assessment of participation enjoyment | The intervention had a significant impact on the participants' ability to identify emotional expressions, such as angry, sad, and happy, from static images of faces, coupled with decreases in reaction time from pre- to post-intervention assessments and follow-up. | | The intervention had a positive impact on the participants' social skills, self-esteem, social competence, and overall quality of life. Also, their satisfaction ratings for reading, painting, and knitting skills, which were either maintained or increased at the 8-month post-intervention follow-up | |
| Agnihotri et al. (2014) | Canada | 13-16 | 20% F | 5 | Acquired brain injury | Pre and post comparison; group comparison | The interventions involved theatre-based activities such as vocal exercises, improvisational acting, and story building, among others. It lasted for four weeks, with daily sessions of four hours each | Emotion discrimination task | | PPIC, Rosenberg Self-Esteem Scale | The improvements were observed in accuracy and reaction time in identifying emotional expressions and emotional regulation | | The positive outcome included the improvements in various social skills such as communication, cooperation, and in overall social functioning | |
| Corbett et al. (2011) | USA | 6-17  Aut M=11.30  (SD=3.98)  Typ M=13.86  (SD=3.49) | 31.25% F | 16 | Autism Spectrum or pervasive developmental disorder versus typically developing children | Pre and post comparison; Group comparison | Social Emotional NeuroScience Endocrinology (SENSE) theatre program involved participants attending majority of 38 rehearsals and 6 performance dates over 3 months. | NEPSY Affect Recognition (Korkman et al., 2007) | | NEPSY Memory for Faces (Korkman et al., 2007) NEPSY Theory of Mind (Korkman et al., 2007); The Social Responsiveness Scale (SRS; (Constantino and Gruber, 2005); The Stress Survey Schedule for Persons with Autism and Other Developmental Delays (SSS; (Groden et al., 2001); The Short Sensory Profile (SSP; (Dunn, 1999); Adaptive Behaviour Assessment System (ABAS; (Harrison and Oakland, 2000a); Basal levels of salivary cortisol 3pre and 3post; Oxytocin collected pre and post | The identification of facial expressions did not reach statistical significance | | ASD participants showed elevated cortisol at the  beginning of the initial rehearsal, which declined over time. It is likely that these findings reflect simple habituation. The effect size suggested that participants showed some improvement in face identification and ToM skills in response to the treatment. Suggesting that exposure to the intervention contributed to some improvement in social perception | |
| Eschenauer et al. (2023) | France | 10.5 | 0% F | 1 | Neurodevelopmental disorder (NDD) characterised by a limitation in cognitive functioning and adaptation capacities. | Pre and post comparison | Performative theatre workshops divided into 8 sessions of 2hrs over 4 months. Complimented by classroom language activities twice a week in sessions of 30min–1hr. | Cognitive-emotional skills (LEAS-C; (Bajgar et al., 2005). | | Executive functions (BRIEF; (Gioia et al., 2013); Creativity; Embodied Language; Oral production | Analysis showed a clear progression in emotional awareness of self and others | | Participants’ scores improved on all indices of the BRIEF measure. Levels of creativity and engagement in activities increased. Oral production increased. | |
| Horwitz et al. (2010) | Sweden | M=53 | 100% F | 7 | Fibromyalgia | Pre and post comparison | Theatre-related method (TRM), 3-month and 2-hour per week, included training in body and voice expression and acting out a drama onstage with professional actors. | Video interpretation technique: patient interpreted herself as she appeared on the videotape and used a 10-point scale to evaluate the intensity of emotional expression in each film. | | Self-rated health and pain | Theatre can be a useful tool for fibromyalgia patients to express their emotions and improve their self-rated health. | | The patients participated in the TRM intervention had a greater reduction in pain perception and their self-rated health improved, compared to those who only watched the play. | |
| Keightley et al. (2018) | Canada | 9-14 | Not mentioned | 3 | Fetal alcohol spectrum disorder (FASD)-related diagnosis | Pre and post comparison | The arts-based theatre training led by a professional theatre artist and educator. It included voice work, breathing, movement, physical warm-up, character development, three-dimensional awareness, group dynamics, story development, mask work, circus skills training, and clowning. It processed 5 days a week for 4 hours over a period of 4 weeks | A self-reported scale of 1 to 10 to be able to communicate their feelings and emotional needs. | | A qualitative research design: participants’ confidence improved | The intervention help express their feelings and improved their emotion expression | | Theatre-based intervention helped increase self-confidence, improve social communication and engagement, and foster a sense of community among the participants. | |
| Mele et al. (2019) | Italy | 47-49 | 28.7% / 31.7% F | 42/26 | Schizophrenia | Pre and post comparison; group comparison | Theatre activities, each patient was supported by a professional educator and consisted of regular participation in rehearsals for the shows and public performances in regional theatres, as well as in national and international tours | To show participants a series of faces and ask them to identify the emotion being expressed. | | -- | The results suggest that drama therapy may be an effective intervention for improving social cognition deficits in individuals with schizophrenia, specifically in the area of facial expression categorization. | | -- | |
| Pires et al. (2020) | Portugal | 16-18 (M = 16.6; SD = 0.8). | 40% F | 5 | Anxiety problems, specifically translated in interpersonal relationships | Pre and post comparison | Therapeutic Mask intervention tool for psychodrama group which was led by an experienced and trained director. It lasted 8 weekly and 90 minutes for each. | Helpful Aspects of Therapy (HAT, (Llewelyn et al., 1988) and the Clinical Change Interview (CCI, (Elliott et al., 2001). | | -- | The adolescents reported improvements in emotional expression and regulation as a result of the intervention. | | The intervention was successful in establishing a positive and supportive therapeutic relationship; reported improvements in interpersonal communication skills, more involvement in day-to-day tasks, and feeling freer to act without feeling judged. | |
| Robinson and Kalawski (2022) | UK | 16-29 | 45.5% F | 11 | Autism spectrum or Asperger Syndrome | Pre and post comparison | The step-out technique in emotion-focused therapy is a tool used in emotion regulation that involves ending each emotional reproduction by at least three slow, regular, and deep, full breathing cycles followed by a total relaxation of the facial muscles and a change in posture. | Client-emotional processing scale for autism spectrum (Robinson and Elliott, 2016) | | - | The intervention helped participants with autistic process shift their attention from an externalised to an internalised process, and to recognise, express, and regulate their internal states. | | The training also greater self-awareness | |
|  |  |  |  |  |  |  |  |  | |  |  | | Reducing the respiratory rate and the concentration of IL-6, an inflammatory response | |
| Tang et al. (2020) | USA | 15-25 | 46% F | 36 | Clinical risk (CR) for psychosis | Pre and post comparison; group comparison | Theatre Improvisation Training to Promote Social Cognition (TIPS) involves 18 weekly 2-hour group sessions led by a theatre director and actor-assistant. Participants engage in collaborative acting and improvisation exercises of varying degrees of complexity, intensity of affect, and interpersonal demand. | Penn computerized neurocognitive battery (CNB, (Moore et al., 2015) | | Clinical Assessment Interview for Negative, Symptoms (CAINS), Structured Interview for Prodromal Syndromes, Global Assessment of Functioning (GAF), Penn Computerized Neurocognitive Battery (CNB), and Penn Age Differentiation Test: | TIPS has no effect on facial emotion processing | | TIPS may improve positive and negative psychosis-spectrum symptoms and Global Assessment of Functioning (GAF) | |
| van Huisstede et al. (2024) | USA | 3-5 (M =50.71 months; SD = 6.44) | 43% F | 196 | Preschoolers, 10% identified as having special needs or disabilities | Pre and post comparison; group comparison | Drama-based instruction (DBI) as part of the EYEPlay program paired professional teaching artists with preschool teachers. Students were expected to experience at least 18 DBI story times but only participated in at least 15 due to the COVID-19 pandemic. Each session begins with discussing drama word or social phrase and encourages children to embody story characters by considering their thoughts and feelings, pantomiming actions and generating new ideas. Children are also given the opportunity to respond physically to reflection questions. | Story Recall Measure (SRM), including the frequency of target emotion words used. Video and audio recording were coded using Embodied Coding System (ECS; (Bernstein et al., 2024) | |  | Intervention children show increased embodied behaviour, especially gestures than the control group. Younger children in intervention also used more emotion words in prompted recall compared to control group. However, there were no overall group differences in free or prompted recall of character feeling states. Embodied behaviours, including facial expressions and vocal changes were positively associated with emotion word production across groups. | | Older students recalled story character feelings more often than  younger students | |
| **Occupational Scenes** | |  |  |  |  |  |  |  | |  |  | |  | |
| Albal et al. (2021) | Turkey | M=35.54 (SD=9.03) | 96% F | 26 | Nurses working at the acute and chronic psychiatric clinics of a psychiatric hospital | Group comparison | The study used psychodrama to help the experimental group gain role flexibility, understand each other's emotions, recognize their own emotions through catharsis, and develop communication skills. The psychodrama lasted two hours on each Thursday for eight weeks. In total, 16 hours were led by a certified psychodrama therapist and a co-therapist | Levels of Emotional Awareness Scale (LEAS, (Kuzucu, 2008); | | The Communication Skills Inventory (CSI, (Ersanlı and Balcı, 1998) to evaluate communication skills and a demographic characteristics questionnaire | Compared with the control group, the psychodrama helped the experiment group better recognise and express their emotions, understand themselves and others, and connect with their own feelings and thoughts better. | | Compared with the control group, the psychodrama improved the communication skills of the experiment group. | |
| Briones et al. (2022) | Spain | M=18.82 (SD=3.07) M=19.16 (SD=3.46) | S1:75.7%F S2:77.6%F | S1:280 S2:626 | Initial / student teacher | Pre and post comparison | Used Creative Drama (CD) and Forum Theatre (FT) to work on conflicts faced by teachers and to enhance students’ social, emotional, and ethical (SEE) skills, which was a one-semester course (approximately 3 months). | Cognitive and affective empathy (Pérez and Pinto, 2009) | | A measure of empathy to test the factors of perspective-taking, empathic stress, and empathic joy.  A 22-item scale to assess the extent to which learners applied the SEE skills education in their behaviour both within the classroom and in the outside world. | The use of CD and FT can have a positive impact on the empathy for the factor of emotional understanding. | | The intervention had a positive impact on participants' SEE skills, both within the classroom and in their broader lives, while it did not find differences in other factors of empathy such as perspective-taking, empathic stress, and empathic joy following the intervention. | |
| Del Vecchio et al. (2022) | USA | Freshmen and Sophomore | Not mentioned | 225 | Nursing students | Pre and post comparison | 2 workshops (non-verbal & verbal communication) were based on classic acting exercises and designed to develop core communication and interpersonal skills. The workshop varied in length from 30 to 90 minutes, as part of the nursing courses in which students learn to interview patients. | 5-point Likert scale (sensitivity to emotions expressed by others). | | 5-point Likert scale (self-awareness, observation, teamwork, flexibility, nonverbal and verbal communication, trust, mindfulness, body language awareness and active listening). | Greater than 85% agreed or strongly agreed that the workshop developed their sensitivity to emotions expressed by others | | The workshop improved their self-awareness, observation, teamwork, flexibility, nonverbal and verbal communication, trust, mindfulness, body language awareness and active listening. | |
| Firing et al. (2022) | Norway | -- | Not mentioned | 14 | Royal Norwegian Air Force Academy students | Pre and post comparison | Theatre-based leadership development programs: (1) Yourself, (2) Relational and social knowledge, and (3) Leadership in the Air Force. It was a nine-week course. | Eight in-depth interviews | | -- | The cadets acquired experiences of how emotions can influence followers in relational contexts, and their acting involved emotional awareness and regulation by means of relational processes with their co-actors and the audience. | | The findings underscore that authenticity, vulnerability, and emotional expression in theatre-based leadership development enhance skills and contribute to impactful learning experiences. | |
| Sisman and Buzlu (2022) | Turkey | 19-21 | 89.2% F | 120 | Nursing students | Group comparison | Emotion-Focused Training Program (EFTP): psychodrama techniques was used, such as sociometry and doubling, role-play, and mirroring. The intervnetion consisted of 10 sessions, each lasting 1.5-2 hours, once a week. | The Emotional Expression Scale (Kuzucu, 2011): verbally and nonverbally. The Levels of Emotional Awareness Scale | | -- | EFTP improved emotion expression skills. Moreover, improved emotion recognition and expression persisted six months post-program during the follow-up assessment. EFTP also improved emotional awareness skills. | | -- | |
| **Children/School Students** | | | | |  |  |  | |  |  |  | |  | |
| Celume et al. (2020) | France | 9-11 | Not mentioned | 126 | -- | Pre and post comparison | Drama Pedagogy Training (DPT): children were asked to create and play a scene with their faces and bodies while the other half of the class had to guess what was going on in the scene, giving details and explanations of their guessing. Collective supportive game (CSG, control group's active intervention): children are divided into two groups, one half has to deliver a message (scarf) and combatants have to avoid the message to be delivered by stealing the message. Total 6 sessions and each lasted 60-70 minutes. | Reading the Mind in the Eyes Test (RMET-G), the ability to infer mental states from facial expressions (Baron-Cohen et al., 1997) | | Prisoner's Dilemma task: Each couple of children are partners in a robbery and have to decide if betray his/her partner to save him/herself or not. The objective of the game is to stay in prison the less time that is possible. | The intervention can promote children's socio-emotional competencies, such as theory of mind and collaborative behaviour. | | -- | |
| Celume and Zenasni (2022) | France | 9-10, 4th grader (M=9.78) | 56% F | 9 | -- | Pre and post comparison | DPT: Each couple of children are partners in a robbery and have to decide if betray his/her partner to save him/herself or not. The objective of the game is to stay in prison the less time that is possible. At the end of each session, a facilitator asked guided questions to help participants thinking. Toal 4 sessions and each lastsd 60-70 minutes. | RMET-G, French version (Baron-Cohen et al., 1997) | | Self-Assessment Manikin scale (SAM): an adapted 9-point scale assessment measuring emotion arousal ; Evaluation of Creative Potential (EPoC): measures the fluency of ideas, resulting in a main divergent thinking score that includes flexibility and originality (Lubart et al., 2011). | A positive effect of the drama pedagogy program on the children's mood but did not show significant improvement in ToM after the training. Also, the observation test showed that the participants spent most of their time discussing socio-emotional themes. | | Improvement in divergent thinking, integrative thinking, and mood, but no significant difference in creativity | |
| Gürle (2018) | Turkey | 10-12 years old | 50% F | 10 | Syrian refugee children | Pre and post comparison | The intervention was a creative arts and drama workshop, made up of 5 2-hour sessions over 2 weeks, involving storytelling, image theatre, improvisation, role play, puppetry, miming, masques and character work. It was led by one lead-facilitator and two assistants and aimed to improve identification and expression of emotions and their communication skills. | The study was an exploratory study that used participant observations of what the children drew and responded to certain statements. | |  | The intervention provided evidence for improved ability to empathise with others and enhanced emotional expression through words or drawings. | | It was found that children became more confident, talkative and willing to engage. Children were able to reconsider their coping mechanisms and there was also more opposite-sex play and interaction by the end of the workshop, mainly initiated by the girls. | |
| Kosma et al. (2024) | USA | Undergraduate M=20.43 | 85.71% F | 7 | -- | Pre and post comparison | Undergraduate students enrolled in one semester long physical theatre (PT) class. Two 1.5hr sessions a week. Participants were interviewed at the start of the semester and at the end. | Audiotaped, structured in-person interviews by first author. Themes and subthemes were identified in the transcripts. | | -- | Participants stated that PT allowed them to unlock their bodies and freely express their emotions. Some participants stated they can express more emotions through movement then they could before PT (not just happy and sad). | | Participants found they had better body-mind connection following PT, as they entered a ‘flow-state’. Participants found they paid more attention to the body language of others following PT and had more confidence in communicating with classmates. | |
| Nikiforidou and Stack (2019) | UK | 3-4 | Not mentioned | 33 | -- | Pre and post comparison | Two conditions: empathic and non-empathic. Each session has three phases: Phase 1 was the read-aloud of the story and phase 2 was the enactment of the story through the perspective of one character per session; namely, session 1: little red riding hood, session 2: grandma, session 3: woodcutter and session 4: wolf. During phase 2, the facilitator would encourage the narration and acting out of the story by addressing for each character four critical moments/dilemmas (Table 2). In phase 3, the children and the facilitator would discuss and reconsider aspects of the story through discussion and free play with ﬁgurines of the story-characters. Total 4 sessions and each lasted 30 minutes. | Video recordings: observational instrument of motor skills (OSMOS, (Castañer et al., 2009) | |  | Children who participated in the empathic condition would use emotions more frequently in their narratives compared to children who in the non-empathic condition. Children were able to verbalise or show more emotion expression when they were encouraged by the facilitators. | | -- | |
| Rousseau et al. (2012) | Canada | 12-18 | 55% F | 55 | -- | Pre and post comparison; group comparison | Each session includes a warm-up period composed of theatrical exercises and of a language awareness activity which also uses dramatization. In the second half of the sessions, stories contributed by the youth are explored and played out in small groups, using either nonverbal expression or the spoken language of their choice. Total 12 sessions (1 session per week) and each lasted 90 minutes. | Participant observations and feedback from teachers and students | | Interviewed about the SES, promigratory experience and perceived language skills, The Strength and Difficulty Questionnaire (SDQ): emotional and behavioural difficulties | Facilitated their expression of feeling | | Overall SDQ scores did not change, but the impairment scores of intervention group (but not control group) decreased. | |

# References

Abdulhaq, B., Al-Khayat, A., Hammouri, M., Raie, J., Eid, A., and Dardas, L. (2025). Integrative art and drama therapy for children with behavioural and emotional challenges: a feasibility study in Jordan. *International Journal of Art Therapy***,** 1-11.

Agnihotri, S., Gray, J., Colantonio, A., Polatajko, H., Cameron, D., Wiseman-Hakes, C., et al. (2012). Two case study evaluations of an arts-based social skills intervention for adolescents with childhood brain disorder. *Dev Neurorehabil* 15(4)**,** 284-297. doi: 10.3109/17518423.2012.673178.

Agnihotri, S., Gray, J., Colantonio, A., Polatajko, H., Cameron, D., Wiseman-Hakes, C., et al. (2014). Arts-based social skills interventions for adolescents with acquired brain injuries: five case reports. *Dev Neurorehabil* 17(1)**,** 44-63. doi: 10.3109/17518423.2013.844739.

Albal, E., Sahin-Bayindir, G., Alanli, O., and Buzlu, S. (2021). The effects of psychodrama on the emotional awareness and communication skills of psychiatric nurses: A randomized controlled trial. *The Arts in Psychotherapy* 75. doi: 10.1016/j.aip.2021.101826.

Bajgar, J., Ciarrochi, J., Lane, R., and Deane, F.P. (2005). Development of the levels of emotional awareness scale for children (LEAS‐C). *British Journal of Developmental Psychology* 23(4)**,** 569-586.

Bar-On, R., and Parker, J. (2000). The bar-on emotional quotient inventory: Youth version (EQ-i: YV). *Technical manual*.

Baron-Cohen, S., Jolliffe, T., Mortimore, C., and Robertson, M. (1997). Another advanced test of theory of mind: evidence from very high functioning adults with autism or asperger syndrome. *J Child Psychol Psychiatry* 38(7)**,** 813-822. doi: 10.1111/j.1469-7610.1997.tb01599.x.

Baron-Cohen, S., and Wheelwright, S. (2004). The empathy quotient: an investigation of adults with Asperger syndrome or high functioning autism, and normal sex differences. *J Autism Dev Disord* 34(2)**,** 163-175. doi: 10.1023/b:jadd.0000022607.19833.00.

Baron-Cohen, S., Wheelwright, S., Hill, J., Raste, Y., and Plumb, I. (2001). The “Reading the Mind in the Eyes” Test revised version: a study with normal adults, and adults with Asperger syndrome or high-functioning autism. *The Journal of Child Psychology and Psychiatry and Allied Disciplines* 42(2)**,** 241-251.

Baron‐Cohen, S., Wheelwright, S., Hill, J., Raste, Y., and Plumb, I. (2003). The “Reading the Mind in the Eyes” Test Revised Version: A Study with Normal Adults, and Adults with Asperger Syndrome or High‐functioning Autism. *Journal of Child Psychology and Psychiatry* 42(2)**,** 241-251. doi: 10.1111/1469-7610.00715.

Bates, D. (2010). lme4: Linear mixed-effects models using S4 classes. R package version 0.999375-37. [*http://www*](http://www)*. r-project. org*.

Beauvais, A., Atli Özbaş, A., and Wheeler, K. (2019). End-of-life psychodrama: influencing nursing students' communication skills, attitudes, emotional intelligence and self-reflection. *Journal of Psychiatric Nursing* 10(2).

Bedell, G.M. (2004). Developing a follow-up survey focused on participation of children and youth with acquired brain injuries after discharge from inpatient rehabilitation. *NeuroRehabilitation* 19(3)**,** 191-205.

Bedell, G.M., and Dumas, H.M. (2004). Social participation of children and youth with acquired brain injuries discharged from inpatient rehabilitation: A follow-up study. *Brain injury* 18(1)**,** 65-82.

Bernstein, K.A., van Huisstede, L., Marley, S.C., Gao, Y.B., Pierce-Rivera, M., Ippolito, E., et al. (2024). Gesture like a kitten and you won't forget your tale: Drama-based, embodied story time supports preschoolers’ narrative skills. *Early Childhood Research Quarterly* 66**,** 178-190.

Bloch, S., Orthous, P., and Santibañez-H, G. (1987). Effector patterns of basic emotions: A psychophysiological method for training actors. *Journal of Social and Biological Structures* 10(1)**,** 1-19.

Bradley, M.M., and Lang, P.J. (1994). Measuring emotion: the self-assessment manikin and the semantic differential. *Journal of behavior therapy and experimental psychiatry* 25(1)**,** 49-59.

Briones, E., Gallego, T., and Palomera, R. (2022). Creative Drama and Forum Theatre in initial teacher education: Fostering students’ empathy and awareness of professional conflicts. *Teaching and Teacher Education* 117. doi: 10.1016/j.tate.2022.103809.

Briones, E., Gómez-Linares, A., Palomera, R., and Carmo, M. (Year). "Teaching in values in higher education: innovation by online dialogue between students from different universities", in: *Proceedings of the International Conference on Education and New Developments*: WIARS), 194-198.

CASEL (2003). *Safe and sound: An educational leader's guide to evidence-based social and emotional learning (SEL) programs.* ERIC Clearinghouse.

Castañer, M., Torrents, C., Anguera, M.T., Dinušová, M., and Jonsson, G.K. (2009). Identifying and analyzing motor skill responses in body movement and dance. *Behavior Research Methods* 41(3)**,** 857-867.

Celume, M.-P., and Zenasni, F. (2022). How perspective-taking underlies creative thinking and the socio-emotional competency in trainings of drama pedagogy. *Estudos de Psicologia (Campinas)* 39. doi: 10.1590/1982-0275202239e200015.

Celume, M.P., Goldstein, T., Besancon, M., and Zenasni, F. (2020). Developing Children's Socio-Emotional Competencies Through Drama Pedagogy Training: An Experimental Study on Theory of Mind and Collaborative Behavior. *Eur J Psychol* 16(4)**,** 707-726. doi: 10.5964/ejop.v16i4.2054.

Charmaz, K. (2006). *Constructing grounded theory: A practical guide through qualitative analysis.* sage.

Conners, C. (1973). Rating scales for use in drug studies with children. *Psychopharmacol Bull* 9**,** 24-42.

Constantino, J., and Gruber, C. (2005). Social responsiveness scale (SRS). Los Angeles, CA: Western Psychological Services. Corkum, V., & Moore, C.(1998). The origins of joint visual attention in infants. *Developmental psychology* 34(1)**,** 2838.

Corbett, B.A., Gunther, J.R., Comins, D., Price, J., Ryan, N., Simon, D., et al. (2011). Brief report: theatre as therapy for children with autism spectrum disorder. *Journal of autism and developmental disorders* 41(4)**,** 505-511.

Crowne, D.P., and Marlowe, D. (1960). A new scale of social desirability independent of psychopathology. *Journal of consulting psychology* 24(4)**,** 349.

Davis, M.H. (1980). A multidimensional approach to individual differences in empathy.

Del Vecchio, A., Moschella, P.C., Lanham, J.G., and Zavertnik, J.E. (2022). Acting to teach communication skills to nurses. *Clin Teach* 19(4)**,** 289-293. doi: 10.1111/tct.13489.

Dunn, W. (1999). Short sensory profile. *San Antonio, TX: Psychological Corporation*.

Elliott, R., Slatick, E., and Urman, M. (2001). Qualitative change process research on psychotherapy: Alternative strategies. *Psychological Test and Assessment Modeling* 43(3)**,** 69.

Ersanlı, K., and Balcı, S. (1998). İletişim becerileri envanterinin geliştirilmesi: Geçerlik ve güvenirlik çalışması. *Turkish psychological counseling and guidance journal* 2(10)**,** 7-12.

Eschenauer, S., Tsao, R., Legou, T., Tellier, M., André, C., Brugnoli, I., et al. (2023). Performing for better communication: Creativity, cognitive-emotional skills and embodied language in primary schools. *Journal of Intelligence* 11(7)**,** 140.

Etkin, A., Egner, T., and Kalisch, R. (2011). Emotional processing in anterior cingulate and medial prefrontal cortex. *Trends in cognitive sciences* 15(2)**,** 85-93.

Firing, K., Thorkelsdóttir, R.B., and Chemi, T. (2022). The Theatre of War: leader development between personal identity and person-in-role. *Culture and Organization* 28(3-4)**,** 330-344. doi: 10.1080/14759551.2022.2028146.

Garnefski, N., and Kraaij, V. (2006). Cognitive emotion regulation questionnaire–development of a short 18-item version (CERQ-short). *Personality and individual differences* 41(6)**,** 1045-1053.

Gentzler, A.L., DeLong, K.L., and Smart, R. (2020). Theater majors compared with nonmajors: Investigating temperament and emotion beliefs, awareness, regulation, and perception. *Psychology of Aesthetics, Creativity, and the Arts* 14(3)**,** 301-312. doi: 10.1037/aca0000219.

Gibbs, G. (2012). *El análisis de datos en investigación cualitativa.* Ediciones Morata.

Gioia, G.A., Isquith, P.K., and Guy, S.C. (2013). *BRIEF: inventaire d'évaluation comportementale des fonctions exécutives.* Hogrefe.

Glaser, B., and Strauss, A. (2017). *Discovery of grounded theory: Strategies for qualitative research.* Routledge.

Goldstein, T.R. (2011). Correlations Among Social‐Cognitive Skills in Adolescents Involved in Acting or Arts Classes. *Mind, Brain, and Education* 5(2)**,** 97-103. doi: 10.1111/j.1751-228X.2011.01115.x.

Goodman, A., Patel, V., and Leon, D.A. (2008). Child mental health differences amongst ethnic groups in Britain: a systematic review. *BMC Public Health* 8**,** 1-11.

Gratz, K.L., and Roemer, L. (2004). Multidimensional assessment of emotion regulation and dysregulation: Development, factor structure, and initial validation of the difficulties in emotion regulation scale. *Journal of psychopathology and behavioral assessment* 26**,** 41-54.

Gray, J., Agnihotri, S., Keightley, M., Colantonio, A., James, J., and Morin, S. (2012). An arts-based approach to co-facilitation of a theatre programme for teenagers with acquired brain injury. *Journal of Applied Arts &amp; Health* 2(3)**,** 221-235. doi: <https://doi.org/10.1386/jaah.2.3.221_1>.

Groden, J., Diller, A., Bausman, M., Velicer, W., Norman, G., and Cautela, J. (2001). The development of a stress survey schedule for persons with autism and other developmental disabilities. *Journal of autism and developmental disorders* 31(2)**,** 207-217.

Gross, J.J., and John, O.P. (2003). Individual differences in two emotion regulation processes: implications for affect, relationships, and well-being. *J Pers Soc Psychol* 85(2)**,** 348-362. doi: 10.1037/0022-3514.85.2.348.

Gürle, N.Ş. (2018). Enhancing the awareness of emotions through art and drama among crisis-affected Syrian refugee children in southeast Turkey. *Intervention Journal of Mental Health and Psychosocial Support in Conflict Affected Areas* 16(2)**,** 164-169.

Hamilton, N.A., Karoly, P., Gallagher, M., Stevens, N., Karlson, C., and McCurdy, D. (2009). The assessment of emotion regulation in cognitive context: The Emotion Amplification and Reduction Scales. *Cognitive Therapy and Research* 33**,** 255-263.

Harrison, P., and Oakland, T. (2000a). "Adaptive behavior assessment system: Psychological Corporation San Antonio". TX).

Harrison, P.L., and Oakland, T. (2000b). *Adaptive behavior assessment system.* Psychological Corporation San Antonio, TX.

Höfling, T.T.A., Alpers, G.W., Büdenbender, B., Föhl, U., and Gerdes, A.B. (2022). What’s in a face: automatic facial coding of untrained study participants compared to standardized inventories. *PLoS One* 17(3)**,** e0263863.

Horwitz, E.B., Kowalski, J., and Anderberg, U.M. (2010). Theater for, by and with fibromyalgia patients – Evaluation of emotional expression using video interpretation. *The Arts in Psychotherapy* 37(1)**,** 13-19. doi: 10.1016/j.aip.2009.11.003.

Ickes, W. (2001). *Measuring empathic accuracy.*: Erlbaum.

Jolliffe, D., and Farrington, D.P. (2006). Development and validation of the Basic Empathy Scale. *J Adolesc* 29(4)**,** 589-611. doi: 10.1016/j.adolescence.2005.08.010.

Jurgens, R., Grass, A., Drolet, M., and Fischer, J. (2015). Effect of Acting Experience on Emotion Expression and Recognition in Voice: Non-Actors Provide Better Stimuli than Expected. *J Nonverbal Behav* 39(3)**,** 195-214. doi: 10.1007/s10919-015-0209-5.

Kaya, F., and Deniz, H. (2020). The effects of using psychodrama on the psychological wellbeing of university students. *Perspectives in Psychiatric care* 56(4)**,** 905-912.

Keightley, M., Agnihotri, S., Subramaniapillai, S., Gray, J., Keresztesi, J., Colantonio, A., et al. (2018). Investigating a theatre-based intervention for Indigenous youth with fetal alcohol spectrum disorder: Exploration d'une intervention basee sur le theatre aupres de jeunes Autochtones atteints du syndrome d'alcoolisme foetal. *Can J Occup Ther* 85(2)**,** 128-136. doi: 10.1177/0008417417719722.

Kilinc, S., Kelley, M.F., Millinger, J., and Adams, K. (2016). Early years educators at play: A research-based early childhood professional development program. *Childhood Education* 92(1)**,** 50-57.

Klinge, C., Röder, B., and Büchel, C. (2010). Increased amygdala activation to emotional auditory stimuli in the blind. *Brain* 133(6)**,** 1729-1736.

Klinge, C., Röder, B., and Büchel, C. (2012). Does training or deprivation modulate amygdala activation? *NeuroImage* 59(2)**,** 1765-1771.

Korkman, M., Kirk, U., and Kemp, S. (2007). "Nepsy—second edition (nepsy-ii)". San Antonio, TX: Harcourt Assessment).

Kosma, M., Erickson, N., and Gremillion, A. (2024). The embodied nature of physical theater: artistic expression, emotions, interactions. *Research in Dance Education***,** 1-26.

Krahmer, E., and Swerts, M. (Year). "On the role of acting skills for the collection of simulated emotional speech", in: *Proceedings of the international conference on spoken language processing (Interspeech 2008)*: ISCA), 261-264.

Kring, A.M., Gur, R.E., Blanchard, J.J., Horan, W.P., and Reise, S.P. (2013). The clinical assessment interview for negative symptoms (CAINS): final development and validation. *American journal of psychiatry* 170(2)**,** 165-172.

Kuzucu, Y. (2008). The adaptation of the levels of emotional awareness scale: validty and reliability studies. *Turkish Psychological Counseling and Guidance Journal* 3(29)**,** 51-64.

Kuzucu, Y. (2011). Adaptation of the emotional expression questionnaire: Validity and reliability studies. *Kastamonu education journal* 19(3)**,** 779-792.

Landazabal, M.G. (1995). Intervención en la creatividad: evaluación de una experiencia. *Revista Iberoamericana de Diagnóstico y Evaluación Psicológica* 1(1)**,** 37-62.

Law, M., King, G., King, S., Hurley, P., Rosenbaum, P., Hanna, S., et al. (2004). Children’s Assessment of Participation and Enjoyment and Preferences for Activities of Children.

Law, M., Polatajko, H., Pollock, N., McColl, M.A., Carswell, A., and Baptiste, S. (1994). Pilot testing of the Canadian Occupational Performance Measure: clinical and measurement issues. *Can J Occup Ther* 61(4)**,** 191-197. doi: 10.1177/000841749406100403.

LeDoux, J. (2007). The amygdala. *Current biology* 17(20)**,** R868-R874.

Linscott, R.J., Knight, R., and Godfrey, H. (1996). The Profile of Functional Impairment in Communication (PFIC): A measure of communication impairment for clinical use. *Brain Injury* 10(6)**,** 397-412.

Llewelyn, S.P., Elliott, R., Shapiro, D.A., Hardy, G., and Firth‐Cozens, J. (1988). Client perceptions of significant events in prescriptive and exploratory periods of individual therapy. *British Journal of Clinical Psychology* 27(2)**,** 105-114.

López-Pérez, B., Fernández-Pinto, I., and García, F.J.A. (2008). *TECA: Test de empatía cognitiva y afectiva.* Tea Madrid.

Lubart, T., Besançon, M., and Barbot, B. (2011). *EPOC: évaluation du potentiel créatif.* Hogrefe.

Mackie, D.M., and Worth, L.T. (1989). Processing deficits and the mediation of positive affect in persuasion. *Journal of personality and social psychology* 57(1)**,** 27.

Malec, J.F. (1999). Goal attainment scaling in rehabilitation. *Neuropsychological Rehabilitation* 9(3-4)**,** 253-275.

Meisner, S., Longwell, D., and Pollack, S. (2012). *Sanford Meisner on Acting.* Knopf Doubleday Publishing Group.

Mele, S., Bivi, R., Borra, L., Callegari, V., Caracciolo, S., Tugnoli, S., et al. (2019). Efficacy of theatre activities in facial expression categorization in schizophrenia. *The Arts in Psychotherapy* 63**,** 141-150. doi: 10.1016/j.aip.2018.08.003.

Merlino, J., and Boal, A. (2014). *Juegos para actores y no actores.* Alba Editorial.

Miller, T.J., McGlashan, T.H., Rosen, J.L., Cadenhead, K., Ventura, J., McFarlane, W., et al. (2003). Prodromal assessment with the structured interview for prodromal syndromes and the scale of prodromal symptoms: predictive validity, interrater reliability, and training to reliability. *Schizophrenia bulletin* 29(4)**,** 703-715.

Moore, T.M., Reise, S.P., Gur, R.E., Hakonarson, H., and Gur, R.C. (2015). Psychometric properties of the Penn Computerized Neurocognitive Battery. *Neuropsychology* 29(2)**,** 235-246. doi: 10.1037/neu0000093.

Nikiforidou, Z., and Stack, J. (2019). The wolf was only feeling hungry: emotional understanding and embodied cognition through dramatic play. *International Journal of Early Years Education* 28(1)**,** 50-62. doi: 10.1080/09669760.2019.1685470.

Opdebeeck, L. (2017). "Forum theatre. Psychosocial and conflict transformation training presentation. ". (Ankara, Turkey: International Organization for Migration).

Orzechowicz, D. (2008). Privileged Emotion Managers: The Case of Actors. *Social Psychology Quarterly* 71(2)**,** 143-156. doi: 10.1177/019027250807100204.

Özbek, A., and Leutz, G. (2013). *Psychodrama: Stage Interaction in Group Psychotherapy.* Ankara: Abdülkadir Özbek Psychodrama Institute Publications.

Pérez, B.L., and Pinto, I.F. (Year). "TECA: Test de empatía cognitiva y afectiva", in: *Avances en el Estudio de la Inteligencia Emocional*: Fundación Marcelino Botín), 79-84.

Petersen, D., and Spencer, T. (2016). CUBED. *Language dynamics group*.

Pires, N., Rojas, J.G., Sales, C.M.D., and Vieira, F.M. (2020). Therapeutic Mask: An Intervention Tool for Psychodrama With Adolescents. *Front Psychol* 11**,** 588877. doi: 10.3389/fpsyg.2020.588877.

Ravens-Sieberer, U., and Bullinger, M. (1998). Assessing health-related quality of life in chronically ill children with the German KINDL: first psychometric and content analytical results. *Quality of life research* 7(5)**,** 399-407.

Ritchie, J., and Spencer, L. (2002). "Qualitative data analysis for applied policy research," in *Analyzing qualitative data*. Routledge), 173-194.

Robinson, A., and Elliott, R. (2016). Brief report: An observational measure of empathy for autism spectrum: A preliminary study of the development and reliability of the client emotional processing scale. *Journal of Autism and Developmental Disorders* 46**,** 2240-2250.

Robinson, A., and Kalawski, J.P. (2022). Experiences of the step-out technique in emotion-focused therapy for clients with autistic process. *Person-Centered & Experiential Psychotherapies* 22(3)**,** 265-282. doi: 10.1080/14779757.2022.2115941.

Rojas-Bermúdez, J., and Moyano, G. (2012). Enmascararse/Desenmascararse. Las máscaras en sicodrama. *Actualizaciones en Sicodrama***,** 113-136.

Rojas-Bermúdez, J.G. (2017). *Teoría y técnica sicodramáticas.* Punto Rojo Libros.

Rosenberg, M. (1965). Society and Adolescent Self-image. *Princeton University*.

Rousseau, C., Armand, F., Laurin-Lamothe, A., Gauthier, M.F., and Saboundjian, R. (2012). A pilot project of school-based intervention integrating drama and language awareness. *Child Adolesc Ment Health* 17(3)**,** 187-190. doi: 10.1111/j.1475-3588.2011.00629.x.

Sales, C., Gonçalves, S., Silva, I., Duarte, J., Sousa, D., Fernandes, E., et al. (Year). "Portuguese adaptation of qualitative change process instruments [Paper presentation]", in: *Annual Meeting of the Society for Psychotherapy Research, Funchal, Madeira, Portugal*).

Schmidt, I., Rutanen, T., Luciani, R.S., and Jola, C. (2021). Feeling for the Other With Ease: Prospective Actors Show High Levels of Emotion Recognition and Report Above Average Empathic Concern, but Do Not Experience Strong Distress. *Front Psychol* 12**,** 543846. doi: 10.3389/fpsyg.2021.543846.

Sisman, F.N., and Buzlu, S. (2022). The impact of an emotion-focused training program on nursing students' emotional awareness and expression: A randomized placebo-controlled study. *Perspect Psychiatr Care* 58(1)**,** 197-205. doi: 10.1111/ppc.12810.

Speilberger, C., Gorsuch, R., and Lushene, R. (1970). The state trait anxiety inventory manual. *Palo Alto, Cal.: Consulting Psychologists*.

Sun, J., and Okada, T. (2021). The process of interactive role-making in acting training. *Thinking Skills and Creativity* 41. doi: 10.1016/j.tsc.2021.100860.

Tamir, M., John, O.P., Srivastava, S., and Gross, J.J. (2007). Implicit theories of emotion: affective and social outcomes across a major life transition. *Journal of personality and social psychology* 92(4)**,** 731.

Tang, S.X., Seelaus, K.H., Moore, T.M., Taylor, J., Moog, C., O'Connor, D., et al. (2020). Theatre improvisation training to promote social cognition: A novel recovery-oriented intervention for youths at clinical risk for psychosis. *Early Interv Psychiatry* 14(2)**,** 163-171. doi: 10.1111/eip.12834.

Treadwell, T.W., Leach, E., and Stein, S. (1993). The social networks inventory: A diagnostic instrument measuring interpersonal relationships. *Small Group Research* 24(2)**,** 155-178.

Ü, D. (2013). *Sociometry and Psychodrama* Istanbul: Remzi Publishing.

van der Schalk, J., Hawk, S.T., Fischer, A.H., and Doosje, B. (2011). Moving faces, looking places: validation of the Amsterdam Dynamic Facial Expression Set (ADFES). *Emotion* 11(4)**,** 907-920. doi: 10.1037/a0023853.

van Huisstede, L., Marley, S.C., Bernstein, K.A., Pierce-Rivera, M., Schmidt, A., Millinger, J., et al. (2024). Drama during story time supports preschoolers’ understanding of story character feeling states. *Journal of Early Childhood Literacy***,** 14687984241240413.

Watson, D., Clark, L.A., and Tellegen, A. (1988). Development and validation of brief measures of positive and negative affect: the PANAS scales. *Journal of personality and social psychology* 54(6)**,** 1063.
